# Impact of automated scoring on interpreting performance and self-regulated learning: evidence from a pedagogical experiment

**DOI:** 10.3389/fpsyg.2026.1867463

**Published:** 2026-09-14

**Authors:** Chen Chen, Ting Liu

**Affiliations:** School of Foreign Languages, Central China Normal University, Wuhan, China

**Keywords:** automated feedback, automated scoring, interpreting training, self-regulated learning (SRL), technology-mediated learning

## Abstract

Confronting the challenges of feedback in traditional interpreting training, this study explores how an automated scoring system affects student interpreters’ performance and self-regulated learning (SRL). Guided by Zimmerman’s cyclical model, the study employed a 14-week quasi-experimental design with two intact classes (*N* = 46), with data triangulated through pre- and post-test scores, questionnaires, and semi-structured interviews. The experimental group outperformed the control group at post-test (*d* = 1.03, 95% CI [0.41, 1.64]), with gains concentrated in linguistic accuracy and logical coherence but absent in information fidelity or delivery fluency, pointing to an uneven effect across interpreting dimensions. SRL results reveal an imbalance in both level and function. Although the Execution-and-Monitoring and Evaluation-and-Reflection subscales scored comparatively high, only the former was significantly associated with score gains (*r* = 0.42, 95% CI [0.02, 0.70], *p* = 0.041), suggesting that high self-reported reflection cannot be assumed to be productive reflection. Pre-learning Planning and Emotional Motivation remained comparatively underdeveloped. The interviews specify why. Students routinely compared their scores but rarely analyzed why errors had occurred. Planning reversed direction, deriving from the previous session’s score rather than from analysis of the upcoming task, and emotional responses diverged. These findings support the value of automated scoring for sustaining deliberate practice, while highlighting the need to align teacher feedback more closely, in timing and individuation, with automated feedback, to support planning and emotional regulation. The contribution therefore lies in specifying which phase of the SRL cycle automated scoring reaches, and with what consequences.

## 1 Introduction

Globalization has heightened the demand for interpreting, and extensive after-class practice is essential to developing interpreting skills. In China, MTI students are required to complete at least 400 tape hours of interpreting practice, yet many still fail to develop satisfactory skills even after meeting this benchmark. A major reason is the lack of effective feedback. Without sufficient supply of interpreting faculty, instructors cannot provide timely, substantive feedback to every student on every after-class exercise. Student interpreters have limited real-time awareness of their own performance, so they depend heavily on post-task feedback to reflect on and regulate their learning. Without such feedback, repeated practice tends to reinforce errors rather than correct them.

AI-based automated scoring systems offer a potential solution, but their actual pedagogical impact remains poorly understood, leaving at least two major gaps. On the one hand, few studies track how these tools perform in real-world classrooms, or which dimensions of interpreting competence actually respond to them. On the other hand, although these systems generate rich, multi-dimensional, and immediate data, it remains unclear how students process and act on this information alongside traditional, delayed teacher feedback, and which phase of their self-regulated learning (SRL) automated scoring feedback reaches. Current research often stops at asking “is the score accurate?” rather than “how does this feedback shape learning behavior?”

To address these gaps, this study adopts a quasi-experimental design comparing an experimental group with a control group to examine the effect of automated interpreting scoring. Drawing on Zimmerman’s (2000, 2002) cyclical model, it explores the SRL characteristics and feedback-utilization patterns among students over a 14-week period. By linking performance changes to SRL processes, it aims to offer empirical evidence for designing hybrid, technology-supported interpreting training, addressing the following research questions:

RQ1: How does the use of an automated interpreting scoring system relate to students’ performance compared with conventional practice? Which dimensions respond?

RQ2: What characteristics do students exhibit across the four questionnaire dimensions of SRL after using the automated interpreting scoring system?

RQ3: How do students process and act on system-generated feedback within their SRL cycle?

## 2 Literature review

### 2.1 Automated scoring in interpreting

#### 2.1.1 The development of automated assessment in interpreting

Interpreting quality assessment has traditionally relied on human evaluators, a process that is time-consuming, cognitively demanding, and difficult to scale (Han and Lu, 2021). Against this backdrop, a growing number of scholars have turned to automated interpreting scoring.

Early research concentrated on temporal and acoustic fluency measures (Yu and van Heuven, 2017), and on lexical, semantic, and informational metrics of fidelity (Papineni et al., 2002; Zhang, 2016; Chung, 2020; Ouyang et al., 2021). Later work combined these into multi-feature, multi-dimensional models (Wang and Yuan, 2023; Ma, 2023), alongside a growing number of studies exploring large language models (LLMs). Seeking to establish whether LLMs can substitute for human raters, Han et al. (2025) conducted three sub-studies: a methodological trial on 56 archived English-Chinese bidirectional consecutive interpretations drawn from Lu and Han’s (2023) dataset; a formative-assessment study in which 30 undergraduates interpreted four source speeches over a semester; and a summative study of 168 examination recordings from 84 students interpreting in both directions. LLM scores correlated strongly with human fidelity ratings (*r* > 0.800 for ChatGPT), and students perceived the AI-generated formative feedback significantly more useful than their teachers’. The study thus establishes both statistical and perceptual acceptability, but its outcome measure is perceived usefulness rather than learning gain. Wang and Wang (2025) examined whether LLMs can assess target-language quality, comparing Claude- and GPT-generated ratings against those of two experienced native-Chinese teacher trainers with high inter-rater agreement (ICC = 0.952). Machine–human agreement was only moderate (*r* = 0.69 and 0.62, respectively). In roughly 7.6% of cases, the models rewarded formal, written-style complexity, while human raters gave higher scores to the strategic simplification commonly used in real-time interpreting. Han’s (2026) systematic review of 69 studies found a post-2020 surge in AI-based translation and interpreting assessment, pointing out persistent weaknesses in benchmark reporting, post-hoc explainability, and cross-condition validation.

These developments mainly focus on validating scoring systems against human benchmarks, rather than on their impact in real teaching contexts. It remains unclear whether and how automated scoring affects students’ interpreting performance or learning behavior over time. This is a gap that the present study attempts to addresses.

#### 2.1.2 Core dimensions

Human evaluation of interpreting has long focused on three major quality dimensions: information fidelity, delivery/fluency, and target-language quality. Although they are not always explicitly categorized as such, this structure is implicit in much of the foundational work on interpreting quality (Bühler, 1986; Gile, 1990; Kurz, 1989; Zwischenberger, 2010).

Information fidelity has attracted the most sustained automation effort because it remains the hardest dimension to model. From n-gram-based metrics (Papineni et al., 2002; Popović, 2015) to embedding-based neural metrics (Sellam et al., 2020; Zhang et al., 2020), these metrics have narrowed the gap with human judgment, but have not closed it. Evidence on their relative merit remains mixed (Lu and Han, 2023; Jiang and Zhang, 2025).

Delivery/fluency, defined by speed, breakdown, and repair (Tavakoli and Skehan, 2005), has produced the most replicable automated findings in this field. Temporal and acoustic variables, particularly unfilled-pause measures and speech rate, consistently predict human fluency ratings (Yu and van Heuven, 2017; Yu and Van Heuven, 2021). Training interpretable machine-learning models on human-rated English-Chinese consecutive interpretations, Jiang and Zhang (2025) found that excessively long, uninterrupted runs of speech were associated with lower rather than higher fluency scores, a pattern they read as a marker of cognitive overload (Lennon, 1990; Mead, 2005) rather than of effortless performance. These findings suggest that automated fluency assessment has matured to a point where it can refine, not merely confirm, existing theoretical constructs.

Target-language quality remains the most underexplored dimension. Wang and Wang’s (2025) finding that LLMs systematically overvalue formal register and written-style complexity indicates that its automated assessment is uniquely vulnerable to a mismatch between written, monolingual norms and the communicative reality of interpreted discourse.

The commercial automated scoring system used in the present study reports feedback along fidelity, fluency, accuracy, and coherence. Therefore, it both reflects and complicates this tripartite framework. Fidelity and fluency match traditional criteria, while accuracy and coherence together correspond to target-language quality, with the software separating lexical-grammatical correctness from discourse-level cohesion. Given that target-language quality remains the least understood of the three, this finer-grained breakdown offers a useful lens for examining whether students’ gains (if any) are more noticeable in some sub-dimensions of target-language quality than in others (RQ1).

### 2.2 Automated assessment and self-regulated learning

#### 2.2.1 Feedback and self-regulated learning in language learning contexts

Self-regulated learning (SRL) refers to the cyclical process through which learners set goals, deploy and monitor strategies, and evaluate outcomes. In Zimmerman’s (2000, 2002) model, this cycle comprises three phases: forethought (task analysis, goal setting, and the motivational beliefs that support them), performance (self-control and self-observation during execution), and self-reflection (self-judgment and self-reaction), with the products of reflection feeding into the forethought phase of the next episode. Feedback is pivotal to this process. Theoretical accounts describe SRL as a recursive process in which internal feedback generated through monitoring serves as the primary driver of regulation (Butler and Winne, 1995). External feedback can enhance this process by supporting students’ monitoring, calibration, and reflection, thereby helping them reduce discrepancies between current performance and goals (Hattie and Timperley, 2007; Thurlings et al., 2013).

Empirical work largely supports this view. In a longitudinal randomized field experiment, Bellhäuser et al. (2023) assigned university students to receive individualized process feedback or no feedback, combining daily diary reports of self-regulation with pre- and post-tests. Multilevel models revealed significant feedback effects on goal setting (*b* = 0.19, SE = 0.08, *p* < 0.05), planning, self-efficacy and daily satisfaction (all *b* = 0.19–0.24) and a stronger effect on adherence to self-set time schedules (*b* = 0.40, SE = 0.17, *p* < 0.05). Exploratory analyses revealed functional differences. Confirmative feedback that reinforced existing behavior and transformative feedback that offered concrete strategies for improvement had stronger effects than purely informative feedback (e.g., *b* = 0.30, SE = 0.09, *p* = 0.001 for satisfaction with the study day). In other words, what matters is not simply whether feedback is given but what function it serves. The trait-level pre–post MANOVA yielded a large effect of time (*η^2^* = 0.29) but no feedback × time interaction (*η^2^* = 0.04, *p* = 0.41). Both groups improved on retrospective trait measures, and feedback offered no additional advantage. This reveals a gap between making quick, short-term improvement and developing lasting self-regulatory skills. Reviewing formative-assessment research, Panadero et al. (2017) reached a compatible conclusion that learners differ in how they process and act on feedback according to their existing self-regulatory skill. Feedback can therefore support SRL development, yet its impact rests on the capacity learners bring to it. This interplay is why this study measures self-regulatory processes alongside performance rather than assuming how students self-regulate based on their final results, and why it examines different SRL phases individually instead of relying on a single, overall score.

#### 2.2.2 Automated and technology-mediated feedback and SRL

With technology becoming more important to language education, researchers have increasingly asked whether automated feedback shapes SRL as teacher feedback does. Most evidence comes from automated writing evaluation (AWE). Han et al. (2021) conducted a mixed-methods quasi-experiment with 280 Chinese non-English-major undergraduates. Participants were divided into a control group, an online-course-only group, and an online-course-plus-AWE group (valid *n* = 73, 70, and 72 after data screening). Based on experiment data and follow-up interviews, they found both technology-supported groups outperformed the control on SRL measures, but the incremental contribution of the AWE tool was limited, indicating that automated feedback is not necessarily better than other well-designed technology use. Sari and Han (2024) tested a hybrid approach instead, comparing 45 Turkish EFL majors receiving both automated and teacher feedback with 34 getting only teacher feedback. Pre- and post-test as well as focus-group interviews were conducted. The hybrid condition showed stronger gains in SRL strategy use and self-efficacy, suggesting that automated feedback works best as a supplement to, rather than a replacement for, human feedback. Tian et al. (2022) added a fresh perspective to this. By comparing how students revise writing based on automated, peer, and teacher feedback, they found that automated feedback prompt students to use more cognitive self-regulatory strategies, implying that different feedback sources spark different aspects of SRL.

But not all findings are positive. Wu et al. (2024) ran K-means clustering on data from 31 Chinese educational-technology undergraduates using an automated feedback tool and identified distinct patterns. The same feedback encourages more careful refinement of their work in some learners, while others appear to engage only superficially with the feedback itself. Wu (2026) similarly cautioned that dense automated output can be difficult to act on without teacher mediation. Where feedback is explicitly scaffolded, by contrast, regulation appears to follow. Tran and Ma (2024) trained 20 Vietnamese English majors in SRL-based online peer feedback. Through text analysis of students’ essays and comments plus interviews, they found that structured training shifted students toward more deliberate strategy use. Xu et al.’s (2026) meta-analysis of 35 AI-supported SRL interventions reflects both sides. The overall effect was moderate (*g* = 0.507), but benefits concentrated in the performance phase and showed considerably less traction in the earlier forethought phase. Automated tools thus appear better suited to helping learners act in the moment than to helping them plan beforehand. These studies suggest that automated feedback can meaningfully support SRL, but its benefits depend on how detailed and actionable the feedback is, how learners choose to engage with it, and whether it is supported by human scaffolding. It also implies that evaluation of such tools should be phase-differentiated rather than global, an implication this study builds into its design.

#### 2.2.3 Automated feedback and SRL in speaking and interpreting

Research on automated feedback and SRL in speaking or interpreting remains much scarcer than in the AWE literature, even though automated interpreting assessment has advanced quickly. Jiang and Zhang (2025) developed an explainable machine-learning framework that scores English–Chinese consecutive interpreting on fidelity, fluency, and target-language use, using SHAP-based feature analysis to identify which aspects of a performance drove a given score. They argued that such transparency can support SRL in ways that an opaque overall score cannot, though the claim rests on design rationale rather than on learner data. Wang and Wang’s (2025) LLM study points to the complementary risk. Where automated judgments diverge from professional norms on register, structure, and flow, learners who defer to them may be misled. Two issues follow. First, AWE research reveals the tension between automated feedback and learner over-reliance. In interpreting, such tension worsens, as quality judgments depend on professional norms that are difficult to encode. Second, while research models pursue explainability, most commercial systems, including the one used in this study, remain black boxes. How students navigate that opacity and whether it hinders accurate causal attribution during self-reflection are underexplored in real educational settings.

Closest to the present design, Yang et al. (2025) ran a quasi-experiment on structured peer assessment with 30 interpreting trainees, combining pre- and post-tests of interpreting performance (inter-rater ICC(2,k) = 0.66, 95% CI [0.29, 0.84] at pre-test; ICC(2,k) = 0.56, 95% CI [0.01, 0.80] at post-test) with SRL and engagement measures and student interviews. A mixed ANOVA yielded a very large effect of time [*F*(1, 28) = 127.76, *p* < 0.001, *η*^2^ₚ = 0.82] and a large time × group interaction [*F*(1, 28) = 9.97, *p* = 0.004, *η*^2^ₚ = 0.26]. The experimental group’s gain scores exceeded the control group’s (*t*(28) = 2.54, *p* = 0.017, Hedges’ *g* = 0.90, 95% CI [0.14, 1.65]). Qualitative data indicated improved confidence, reflective thinking, and goal setting. Although the feedback source is human, the study demonstrates that regular, structured feedback can drive SRL gains in interpreter training, and its design provides the methodological template the present study extends to an automated source.

Taken as a whole, these studies point in a consistent direction. Automated interpreting assessment tools are becoming more capable of producing detailed, learner-usable feedback, yet no study has directly examined how such feedback shapes the SRL process within a controlled, quasi-experimental design. Addressing that gap requires a framework capable of specifying which part of the self-regulatory cycle automated feedback reaches.

### 2.3 Theoretical framework of this study

This study adopts Zimmerman’s (2000, 2002) three-phase model of SRL (outlined in Section 2.2.1) as the primary analytical lens to examine how students engage with the automated interpreting scoring system and use its feedback.

Three considerations motivate this choice. First, Zimmerman’s model is the most widely used SRL framework in educational research, which facilitates comparison with existing work. Second, its phases anchor RQ2 and RQ3. Forethought covers planning and goal setting; performance covers self-control and monitoring during execution; and self-reflection corresponds to how students interpret, evaluate, and act on system feedback. Third, since the model is cyclical, it can accommodate the possibility, indicated by Xu et al. (2026), that a feedback intervention reaches some phases but not others, instead of treating SRL as a single global capacity.

Within this framework, each of the system’s four feedback dimensions functions as an input into self-judgment. Students compare observed performance against a dimension’s score (self-evaluation) and may attribute strengths or weaknesses to specific causes (causal attribution) (Zimmerman, 2000, 2002). Whether such judgment translates into self-reaction and subsequent adjustment of goals or strategies distinguishes productive engagement from passive reception. The two structures are cross-cutting rather than parallel. The feedback dimensions describe what aspect of performance is evaluated, whereas the SRL phases describe what kind of self-regulatory process the student engages in. Consistent with Zimmerman’s placement of self-motivation beliefs within forethought (Pintrich, 2000; Zimmerman, 2000, 2002), motivational constructs such as self-efficacy and outcome expectations are treated here as forethought sub-processes rather than as a separate phase. The correspondence between the model’s phases and the questionnaire’s four measurement blocks is specified in Section 3.3.4.

Two narrower tools are introduced later to account for uneven gains across dimensions: Shute’s (2008) taxonomy, which characterizes the system’s output as individualized knowledge of results and of the correct response rather than as elaborated feedback (Section 5.1.1), and Kluger and DeNisi’s (1996) Feedback Intervention Theory, which distinguishes task dimensions by their responsiveness to feedback (Section 5.1.2). As prior research shows that automated feedback engages cognitive strategies more readily than motivational regulation, and that its effects concentrate mainly in the performance phase (Tian et al., 2022; Xu et al., 2026), this study therefore anticipates that engagement will be more apparent in performance-phase behavior than in forethought-phase beliefs. Section 5 examines this claim using both quantitative and interview data.

## 3 Research methodology

### 3.1 Research design

This study adopted a quasi-experimental design to examine the impact of automated interpreting scoring on student interpreters’ performance and SRL. Institutional and administrative constraints made a randomized controlled trial infeasible, so two existing intact classes served as the experimental (*n_1_* = 24) and the control (*n_2_* = 22) groups. Assignment of the two classes to conditions was determined by coin flip before the intervention, in order to avoid systematic allocation bias. Nevertheless, unmeasured class-level confounding variables cannot be fully excluded (Section 6.3).

Over 14 weeks, the experimental group completed their after-class interpreting tasks on the Yunyi Interpreting Quality Assessment System, which provided immediate feedback upon submission, including automated scores, transcriptions, reference renditions, and marked errors. The control group relied on traditional methods, recording their performance on electronic devices and submitting them to the instructor. Both groups received collective feedback in the following week’s class, and both took pre- and post-tests graded by two trained raters. After the intervention, the experimental group filled out a structured questionnaire, and seven students were invited to semi-structured interviews. This mixed-method design allowed a more comprehensive understanding of changes in performance and SRL process.

As the control group lacked immediate, system-generated feedback during the semester, a post-experiment compensation design was implemented to ensure educational equality and ethical standards. Following the 14-week intervention, control group students were granted full access to the Yunyi system. Training sessions were subsequently provided to ensure equitable exposure to the technological benefits.

### 3.2 Participants

Forty-six sophomores majoring in English Translation and Interpreting at a normal university in central China participated in the study. Both groups had already completed the prerequisite course *Interpreting 101* with the same instructor. The same instructor and teaching materials were retained throughout the 14-week experiment to minimize teaching bias.

Baseline equivalence was assessed at the outset to mitigate selection bias. Of the 46 participants, 7 were male and 39 female (experimental group: 4 male, 20 female; control group: 3 male, 19 female). Gender balance (Chi-square test) and pre-test comparability (*t*-test) were examined. The results are reported with the post-intervention outcomes in Section 4.1.1 to ensure a coherent statistical narrative. Between-group comparisons of pre-test sub-scores across the four interpreting dimensions are likewise detailed in Section 4.1.2.

All participants were informed of the study’s goals and assured that their final grades would be unaffected and their personal data kept confidential. Informed consent was obtained before data collection. Since the control group received no system-generated feedback during the semester, all control-group students were granted full access to the Yunyi system, together with training sessions, after the post-test (Section 3.1).

### 3.3 Research process

#### 3.3.1 Pre-test

The baseline was established via a pre-test scored independently by two trained raters. The human rating rubric adopted the assessment framework embedded in the Yunyi system, comprising four weighted criteria: Information Fidelity, 35%; Delivery Fluency, 30%; Linguistic Accuracy, 25%; and Logical Coherence, 10%. Although this framework is the system’s own settings, its four criteria map onto the dimensions used in established interpreting rubrics, which typically distinguish content/fidelity, target-language quality, and delivery (Lee, 2008), and which have been examined in rubric-based and many-facet Rasch validation studies (Han, 2015) and in comprehensive reviews of scale-based assessment practices (Han, 2018; Wu, 2013), with logical coherence treated as a separable component of the content dimension. As Yunyi is a proprietary commercial product, its scoring algorithms and the derivation of these weights are not publicly documented. The rubric was adopted for human scoring not on the strength of its weights, but to maintain construct consistency between human and automated scores. Applying the same construct at pre-test also ruled out scoring disparity as a confounding variable.

Before formal scoring, the two raters jointly rated three pilot recordings and discussed discrepancies until reaching a shared understanding of the four criteria. Recordings were anonymized and presented randomly, and both raters remained blind to student’s group membership and test timing. The mean of raters’ scores was used for each student in subsequent analysis. Inter-rater agreement was assessed by Pearson correlation and ICC(2, k) and is reported together with the post-test values in Section 4.1.1.

#### 3.3.2 Intervention

Before the intervention, a training session was held to familiarize the experimental group with the Yunyi Interpreting Quality Assessment System, an AI-powered system that integrates automatic speech recognition, machine translation, text-to-speech, and automated scoring algorithms. During the orientation, students were introduced to the system’s scoring rubric, the nature of the feedback, and methods for accessing them.

As a proprietary commercial product, the Yunyi system does not disclose its computational formulations. This opacity presents an inherent limitation to the study (Section 6.3). To assess the credibility of its feedback, a pilot validation comparing automated scores with human ratings on 40 recordings was conducted (Section 3.5). The results, particularly the variation in scoring reliability across dimensions, were explicitly communicated to students during the orientation to manage expectations and to frame the system as a formative scaffolding tool rather than a high-stakes evaluator.

Starting from the intervention, both groups followed the standard curriculum. Weekly after-class exercises consisted of approximately one-minute audio materials, alternating between English-to-Chinese (approx. 150 words) and Chinese-to-English (approx. 250 characters) tasks, totaling seven assignments per direction. For the experimental group, assignments were released via Yunyi, with each clip cut into three segments. The control group received the full audio file but was instructed by the same instructor to pause at three points, following the segmentation practice already established in class. Students in both groups were encouraged to practice repeatedly before submitting their best rendition. The conditions therefore differed only in whether students received an immediate, individualized score and diagnostic record after each attempt. Both groups received instructor feedback in the following week’s class.

#### 3.3.3 Post-test

A post-test was conducted after the intervention to assess changes in interpreting performance. Both tests involved Chinese-to-English speeches on general topics of comparable length (pre-test 1′52″/350 characters; post-test 1′43″/340 characters). Propositional density, calculated using CPIDR (Computerized Propositional Idea Density Rater) on the English reference renditions, was examined as an index of textual difficulty comparability and is reported in Section 4.1.1 so that the equivalence of test materials can be evaluated jointly with the performance findings it supports. The same format, procedure, raters, and assessment rubrics were adopted in both tests. Post-test inter-rater agreement and reliability (Pearson’s *r* and ICC) are likewise reported in Section 4.1.1, and the mean of the two raters’ scores served as each student’s final score.

#### 3.3.4 Post-experiment questionnaire

After the post-test, a questionnaire on SRL in the context of automated interpreting scoring was distributed to the 24 experimental-group students. All responses were returned within the given timeframe and confirmed valid. As conceptualized in Section 2.3, this study draws on Zimmerman’s (2000, 2002) three-phase cyclical model, because it suits the process-oriented nature of interpreting training. Trainees prepare for a task, manage real-time cognitive loads during the task, and reflect upon performance afterwards. So the questionnaire was adapted from the Self-Regulation of Learning Self-Report Scale (SRL-SRS; Toering et al., 2012), which was originally designed based on Zimmerman’s model.

The original six dimensions were consolidated into four: Pre-learning Planning, Execution and Monitoring, Evaluation and Reflection, and Emotional Motivation. Self-monitoring and effort were integrated into Execution and Monitoring to capture the synchronous processing and intense cognitive load required during real-time interpreting. Evaluation and reflection were merged because both belong to the self-reflection phase. Empirically, a substantial correlation has been found between them (*r* = 0.58; Toering et al., 2012). Planning was retained as Pre-learning Planning, and Self-efficacy was relabeled Emotional Motivation, reflecting Bandura’s (1997) account of self-efficacy as inseparable from its affective correlates (anxiety, confidence, and emotional reactivity to performance outcomes) rather than a purely cognitive judgment. This label better captures the affective dimension in the automated-feedback context, where individualized, immediately visible scores were expected to have a stronger emotional effect than delayed collective feedback. Items were measured on a five-point Likert scale, ranging from 1 (never/strongly disagree) to 5 (always/strongly agree).

Internal consistency for the overall scale and its four subscales was assessed via Cronbach’s *α* and is reported together with the descriptive results in Section 4.2.1. As is discussed there, the coefficient for Pre-learning Planning fell below the conventional 0.70 threshold. This dimension was nonetheless retained because it is a theoretically indispensable component of Zimmerman’s (2000, 2002) forethought phase. Without it, the results would no longer fully represent the three-phase SRL cycle. Moreover, this subscale comprises only three items, and Cronbach’s *α* is strongly attenuated by test length. Its mean inter-item correlation is 0.34, which falls within the 0.15–0.50 range recommended for a narrowly defined construct (Clark and Watson, 1995), indicating adequate item coherence. Given that low reliability reduces observed correlations, the non-significant association between this dimension and score gains (Section 4.2.2) may partly reflect measurement error rather than the absence of a relationship.

Validity was ensured from two aspects. First, the questionnaire was derived from an established scale and was refined based on the expert feedback from a pedagogy professor and an interpreting training specialist. Their review helped improve item clarity and accuracy. Second, structural validity was examined through item-total and inter-dimension correlations. Given the modest sample size (*N* = 24), formal exploratory or confirmatory factor analysis was not conducted, as such procedures require considerably larger samples for stable parameter estimates (Costello and Osborne, 2005). Correlations evidence was therefore adopted as a more sample-appropriate indicator and is reported alongside the reliability findings in Section 4.2.1.

#### 3.3.5 Post-experiment interview

Seven experimental-group students were selected for semi-structured interviews using stratified purposive sampling based on pre-test scores (two high-, three mid-, and two low-scoring students) to capture experiences across baseline performance levels. These interviews, lasting 15–20 min each and conducted either in person or via online platforms, explored how students planned before a task, monitored performances during it, and reflected afterwards. Psychological factors such as self-efficacy and learning motivation were also discussed. All interviews were conducted in Mandarin Chinese, audio-recorded with consent, and later transcribed verbatim. Excerpts reported here were translated into English by the researcher. Given the modest sub-sample (*N* = 7), the qualitative findings in Section 4.3 are intended to illustrate and help explain patterns observed in the quantitative data, rather than to support generalizable claims about the proportion of students holding a given view. This interpretive stance is maintained throughout the presentation of results.

### 3.4 Data analysis

Quantitative data analysis was conducted using SPSS (Version 31.0.1.0). Baseline equivalence and post-intervention gains were evaluated using Chi-square tests, independent samples *t*-tests and 2 (Group: experimental vs. control) × 2 (Time: pre-test vs. post-test) mixed design ANOVA. ANOVA assumptions were tested (Shapiro–Wilk, Levene’s test, and Box’s *M*), and the results are reported alongside the corresponding findings in Section 4.1. Standardized effect sizes and 95% confidence intervals were calculated throughout, and descriptive statistics were used to map the trends. Inter-rater reliability for both the pilot validation of the automated scoring system (Section 3.5) and the pre- and post-test human ratings (Section 4.1.1) was assessed via Pearson correlation and ICC.

For the questionnaire, internal consistency was assessed with Cronbach’s *α*, and structural validity with item-total and inter-dimension Pearson correlations (Section 4.2.1). Descriptive analysis characterized students’ SRL behaviors in the automated interpreting scoring context. To examine the relationship between SRL and improvement, Pearson correlations were computed between the four SRL dimensions and value-added (post-test minus pre-test) scores, followed by a multiple linear regression analysis entering all four SRL dimensions simultaneously as predictors of score gains. Variance inflation factors (VIF) were examined to rule out problematic multicollinearity given the moderate inter-dimension correlations reported in Section 4.2.1.

Interview transcripts were analyzed using reflexive thematic analysis (Braun and Clarke, 2006, 2019) and coded in NVivo (Version 15.5.2). Coding was conducted by a single researcher following an inductive, data-driven approach. Codes were generated and iteratively refined across multiple passes, then organized into categories and higher-order themes corresponding to the four SRL dimensions. Consistent with the reflexive thematic analysis tradition, which treats coding as an interpretive, researcher-driven process rather than one requiring inter-coder consensus, no second coder or member-checking procedure was employed.

### 3.5 Preliminary validation of the automated scoring system

Since the internal mechanisms of the Yunyi system could not be independently verified (Section 3.3.2), a preliminary criterion check was conducted before the intervention. Forty pilot recordings were independently scored by both the system and the two trained raters who subsequently scored the pre−/post-tests.

The results indicated that the automated scores were generally lower than human scores (Automated: *M* = 53.32, *SD* = 7.17; Human: *M* = 80.40, *SD* = 5.60), reflecting the system’s strict and fine-grained deductions as against the raters’ holistic tolerance of minor errors. Nevertheless, the two sets of total scores had weak to moderate correlation (Pearson *r* = 0.36, *p* = 0.022, 95% CI [0.06, 0.60]; Spearman *ρ* = 0.35, *p* = 0.026, 95% CI [0.04, 0.60]). Dimension-level agreement, however, varied widely (Table 1).

**Table 1:** Pearson and Spearman correlations between automated and human ratings by dimension.

Dimension	Pearson *r*	*p*	95% CI	Spearman *ρ*	*p*	95% CI
Total score	0.36	0.022	[0.06, 0.60]	0.35	0.026	[0.04, 0.60]
Information fidelity	0.12	0.478	[−0.20, 0.41]	0.12	0.471	[−0.21, 0.42]
Delivery fluency	0.45	0.004	[0.16, 0.67]	0.56	<0.001	[0.29, 0.74]
Linguistic accuracy	0.63	<0.001	[0.39, 0.78]	0.65	<0.001	[0.42, 0.81]
Logical coherence	0.79	<0.001	[0.64, 0.89]	0.78	<0.001	[0.61, 0.88]

*N* = 40 pilot recordings. Correlations were computed between automated scores and the mean of two human raters’ scores.

Logical coherence (*r* = 0.79, *p* < 0.001, 95% CI [0.64, 0.89]) and linguistic accuracy (*r* = 0.63, *p* < 0.001, 95% CI [0.39, 0.78]) showed strong correlations with human ratings; delivery fluency (*r* = 0.45, *p* = 0.004, 95% CI [0.16, 0.67]) a moderate one; and information fidelity (*r* = 0.12, *p* = 0.478, 95% CI [−0.20, 0.41]) a weak, non-significant correlation. Agreement was thus weakest on the dimension carrying the greatest weight in the rubric (35 of 100 points) and on the one requiring semantic comparison of propositional content. This is also consistent with the automated speech scoring literature, in which delivery-related constructs align with human raters more closely than content-related ones (Zechner et al., 2009; Chen et al., 2018). This also resolves an apparent paradox in the figures above. The total-score correlation (*r* = 0.36) is lower than that of three of its four constituent dimensions. As fidelity carries the greatest weight (35%) while agreeing least with human raters, it dominates and systematically depresses the composite, so the aggregate score is a weaker indicator than the sub-scores. This is a further reason to treat the system as formative scaffolding rather than as a measurement instrument. Both the baseline differences and the fidelity-dimension limitation were explained to students during the training (Section 3.3.2). This dimensional profile is referred to again in Section 4.1.2 and interpreted in Section 5.1.2.

## 4 Results

### 4.1 Effects on interpreting performance

#### 4.1.1 Overall changes in performance

The two groups were comparable at baseline. Gender distribution did not differ [*χ*^2^ (1) = 0.08, *p* = 0.775, Cramér’s *V* = 0.04]. The experimental group (*M* = 80.06, *SD* = 5.95) and the control group (*M* = 79.55, *SD* = 4.06) were well-matched, with no statistically significant difference observed (*t*(44) = 0.34, *p* = 0.735, Cohen’s *d* = 0.10, 95% CI [−0.48, 0.68]). Shapiro–Wilk tests indicated normally distributed scores in both groups at both time points (*p* > 0.05 for all conditions). Levene’s test confirmed the homogeneity variance for pre-test (*p* = 0.209) and post-test (*p* = 0.465) scores.

The pre- and post-test speeches were closely matched on propositional density (CPIDR: 0.545 vs. 0.537), alongside comparable duration (1′52″ vs. 1′43″), genre, and delivery speed (188 CPM vs. 198 CPM). Therefore, pre-post differences cannot be attributed to differing task difficulty. The two raters agreed closely at both time points (pre-test: *r* = 0.71, *p* < 0.001, ICC(2, k) = 0.82, 95% CI [0.68, 0.90]; post-test: *r* = 0.70, *p* < 0.001, ICC(2, k) = 0.81, 95% CI [0.65, 0.90]). Therefore, their mean was used as each student’s pre- and post-test score.

A 2 × 2 mixed-design repeated-measures ANOVA was then conducted on total scores. With only two levels of the within-subject factor, sphericity was not at issue, and homogeneity of covariance matrices was met (Box’s *M* = 7.68, *p* = 0.063). There was a significant main effect of time [*F*(1, 44) = 46.83, *p* < 0.001, *η*^2^ₚ = 0.52], showing improvement in both groups, and a significant time × group interaction [*F*(1, 44) = 18.00, *p* < 0.001, *η*^2^ₚ = 0.29] (Figure 1). Both values exceed conventional thresholds for a large effect (Cohen, 2013). At post-test the experimental group (*M* = 85.58, *SD* = 5.07) scored higher than the control group (*M* = 80.84, *SD* = 4.01), *t*(44) = 3.50, *p* = 0.001, *d* = 1.03, 95% CI [0.41, 1.64] (Table 2). A sensitivity analysis (G*Power 3.1.9.7) indicated that with *n₁* = 24 and *n₂* = 22 (*α* = 0.05, power = 0.80) the design could reliably detect effects of *d* ≥ 0.85. The observed effect exceeds this threshold, although smaller true effects could not have been detected with confidence.

**Figure 1:**
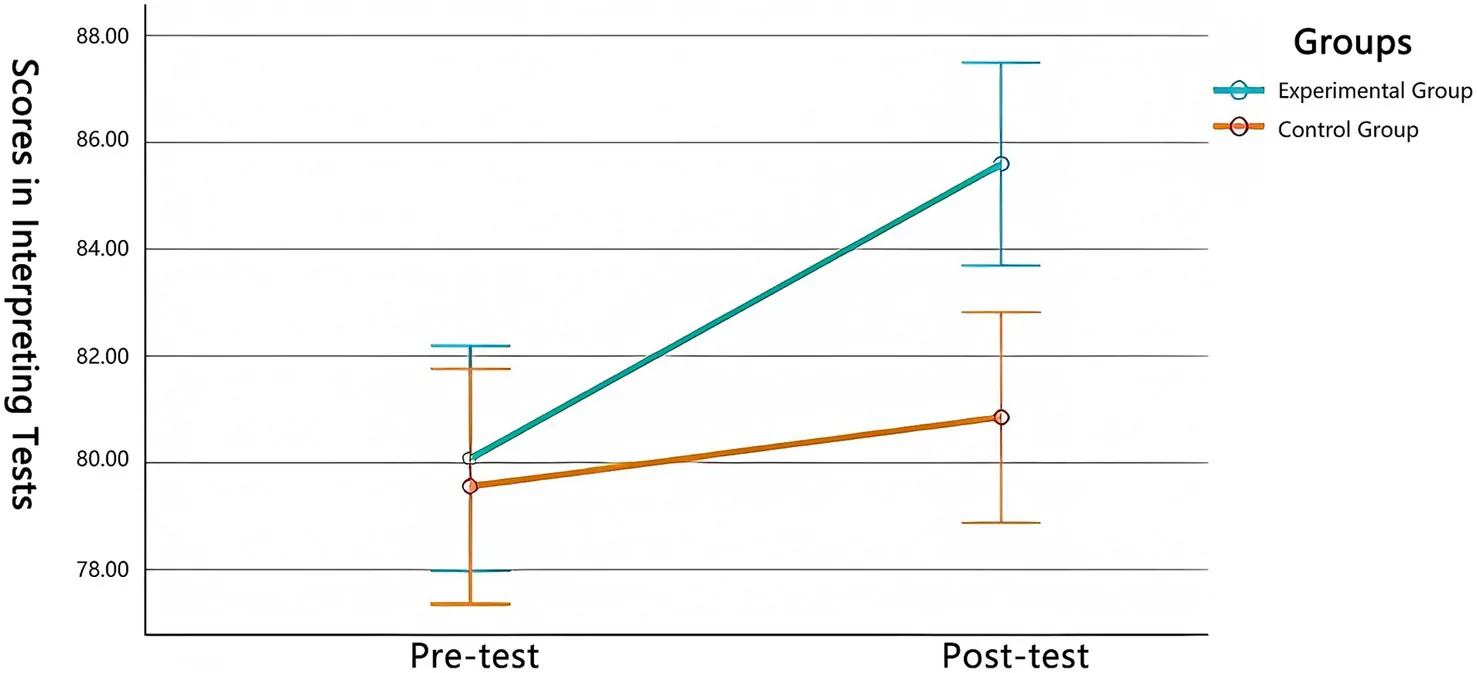
Interaction effect of time and group on pre- and post-test interpreting scores for experimental and control classes. Error bars represent 95% confidence intervals.

**Table 2:** Total test scores by group.

Time	Experimental group *M* (*SD*)	Control group *M* (*SD*)	*t*	*p*	Cohen’s *d*	95% CI
Pre-test	80.06 (5.95)	79.55 (4.06)	0.34	0.735	0.10	[−0.48, 0.68]
Post-test	85.58 (5.07)	80.84 (4.01)	3.50	0.001	1.03	[0.41, 1.64]

Experimental Group *n*_1_ = 24; Control Group *n*_2_ = 22. Unless otherwise noted, df = 44 for both t tests. CI = confidence interval for the mean difference (Experimental - Control).

Figure 2 displays the distribution of total scores for both groups at pre- and post-test. The experimental group’s distribution shifted to the right between tests, whereas the control group’s remained largely unchanged, consistent with the interaction effect reported above.

**Figure 2:**
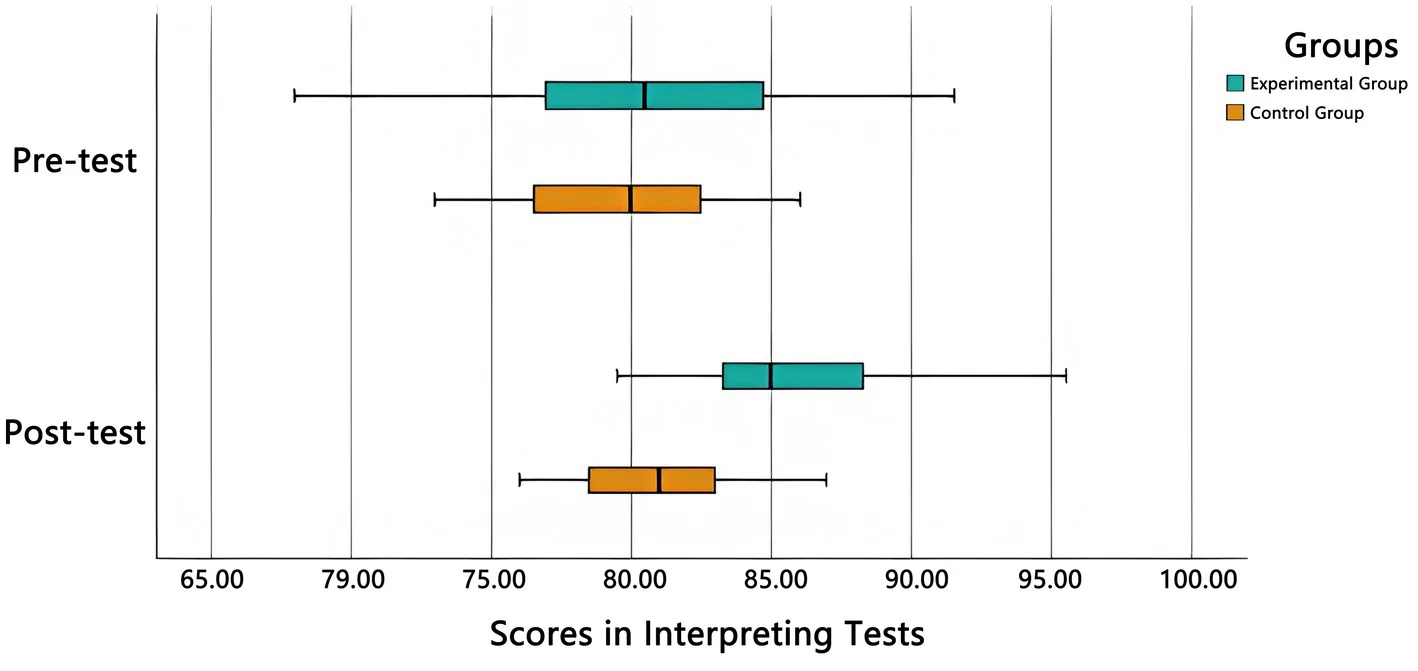
Box plots of pre- and post-test interpreting scores for both groups.

#### 4.1.2 Performance across four dimensions

Pre-test sub-scores did not differ between groups on any of the four criteria (all *p*s > 0.05, all *d*s ≤ 0.20, all 95% CIs including zero; Table 3). Assumptions for the dimension-level ANOVAs were largely satisfied. Levene’s tests were non-significant at both time points for information fidelity, delivery fluency and linguistic accuracy (all *p*s > 0.08), but not for logical coherence at post-test (*p* < 0.001), for which a Welch-adjusted *t* test is reported in Table 3. Box’s *M* was non-significant for fidelity, accuracy and coherence (all *p*s > 0.08), and significant for fluency (*p* = 0.013), where the standard ANOVA was retained given the test’s sensitivity to sample size and the near-equal group sizes.

**Table 3:** Pre- and post-tests sub-dimension scores by group.

Dimension	Time	Experimental *M* (*SD*)	Control *M* (*SD*)	*t*	*p*	Cohen’s *d*	95% CI
Information fidelity	Pre-test	26.13 (4.10)	25.80 (2.75)	0.32	0.753	0.09	[−0.49, 0.67]
	Post-test	26.17 (3.55)	24.89 (2.45)	1.41	0.166	0.42	[−0.17. 0.99]
Delivery fluency	Pre-test	25.90 (1.85)	25.57 (1.32)	0.69	0.496	0.20	[−0.38, 0.78]
	Post-test	26.08 (2.03)	25.93 (1.50)	0.29	0.776	0.08	[−0.50, 0.66]
Linguistic accuracy	Pre-test	21.04 (1.79)	21.18 (1.01)	−0.32	0.748	−0.10	[−0.67, 0.48]
	Post-test	24.25 (1.08)	22.70 (1.13)	4.73	<0.001	1.40	[0.74, 2.04]
Logical coherence	Pre-test	7.00 (2.14)	7.00 (2.33)	0.00	1.000	0.00	[−0.58, 0.58]
	Post-test	9.08 (1.32)	7.36 (2.30)	3.07^a^	0.004	0.93	[0.31, 1.53]

Experimental Group *n*_1_ = 24; Control Group *n*_2_ = 22. Unless otherwise noted, df = 44. CI = Confidence Interval. ^a^Due to unequal variances, Welch’s *t*-test was used for Logical Coherence (post- test), df = 32.80.

The time × group interaction was significant for linguistic accuracy [*F*(1, 44) = 13.20, *p* < 0.001, *η*^2^ₚ = 0.231] and logical coherence [*F*(1, 44) = 5.40, *p* = 0.025, *η*^2^ₚ = 0.109] but not for information fidelity [*F*(1, 44) = 1.42, *p* = 0.240, *η*^2^ₚ = 0.031] or delivery fluency [*F*(1, 44) = 0.099, *p* = 0.755, *η*^2^ₚ = 0.002] (Figures 3–6). The experimental group gained 3.21 points on accuracy (21.04 → 24.25 of 25) and 2.08 on coherence (7.00 → 9.08 of 10), against control-group gains of 1.52 and 0.36 respectively, and was significantly ahead on both at post-test. Fidelity and fluency barely moved in the experimental group (+0.04 and +0.18), while the control group declined on fidelity (−0.91) and gained slightly more on fluency (+0.36).

**Figure 3:**
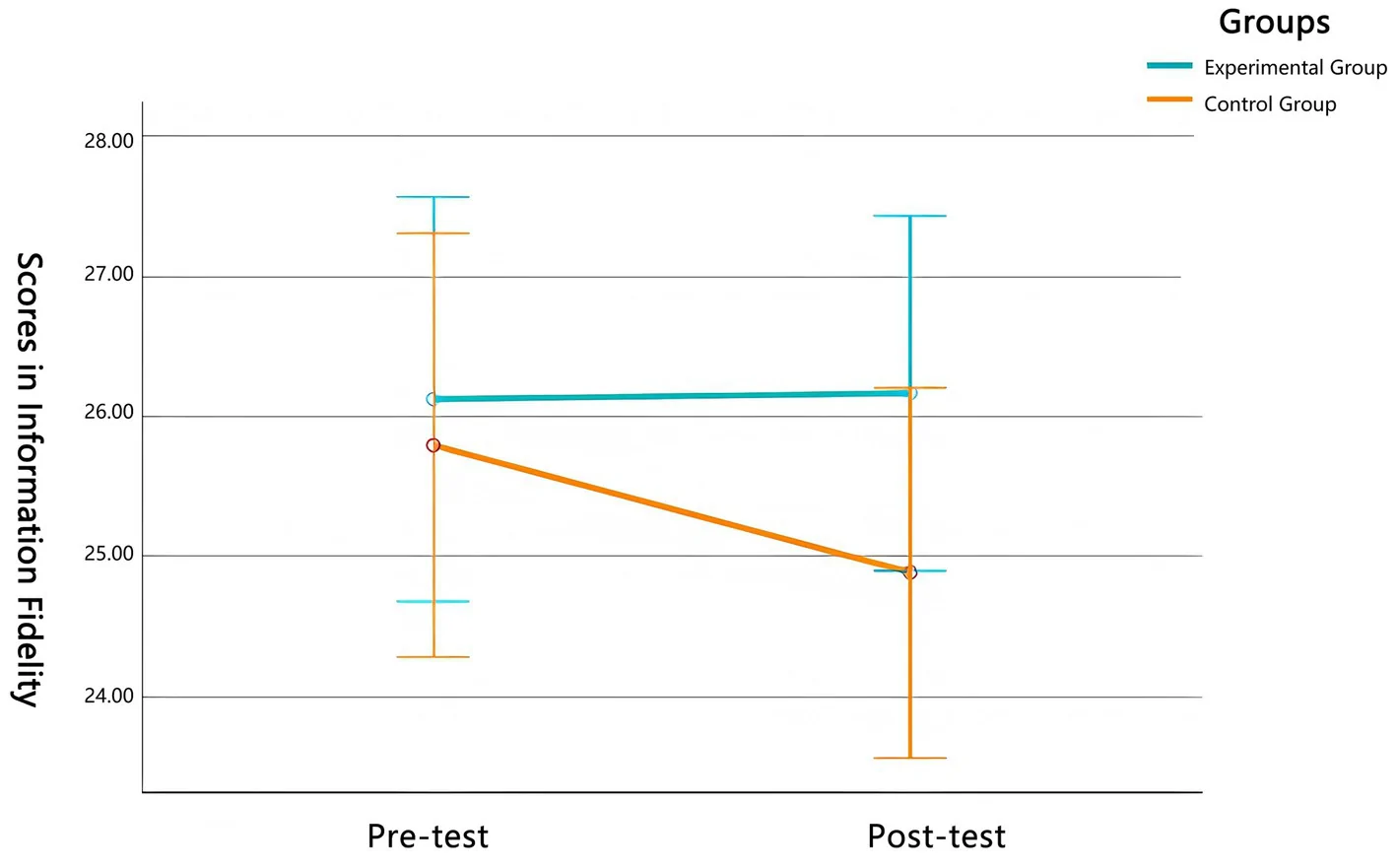
Interaction effect of time and group on information fidelity scores in pre- and post-test interpreting. Error bars represent 95% confidence intervals.

**Figure 4:**
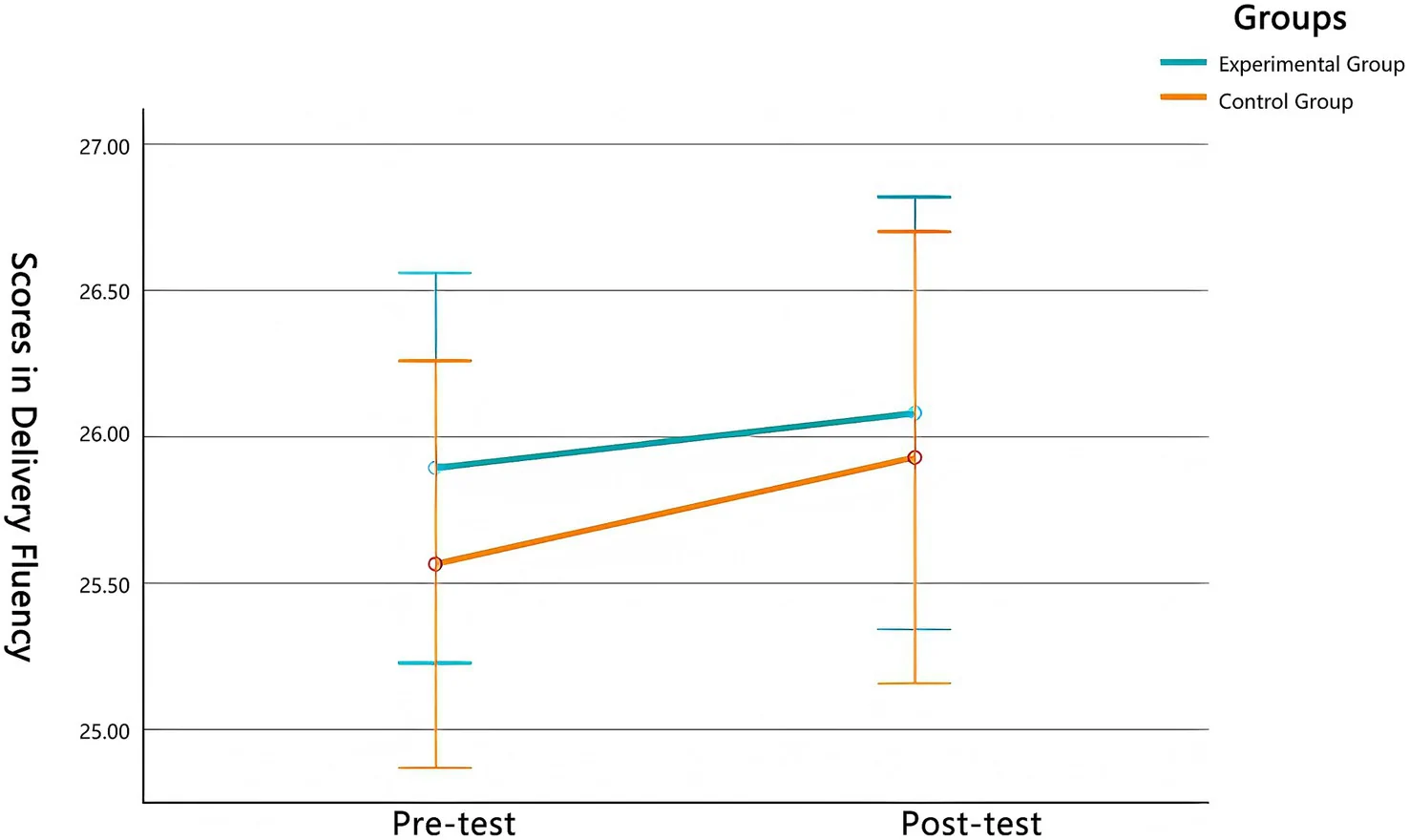
Interaction effect of time and group on delivery fluency scores in pre- and post-test interpreting. Error bars represent 95% confidence intervals.

**Figure 5:**
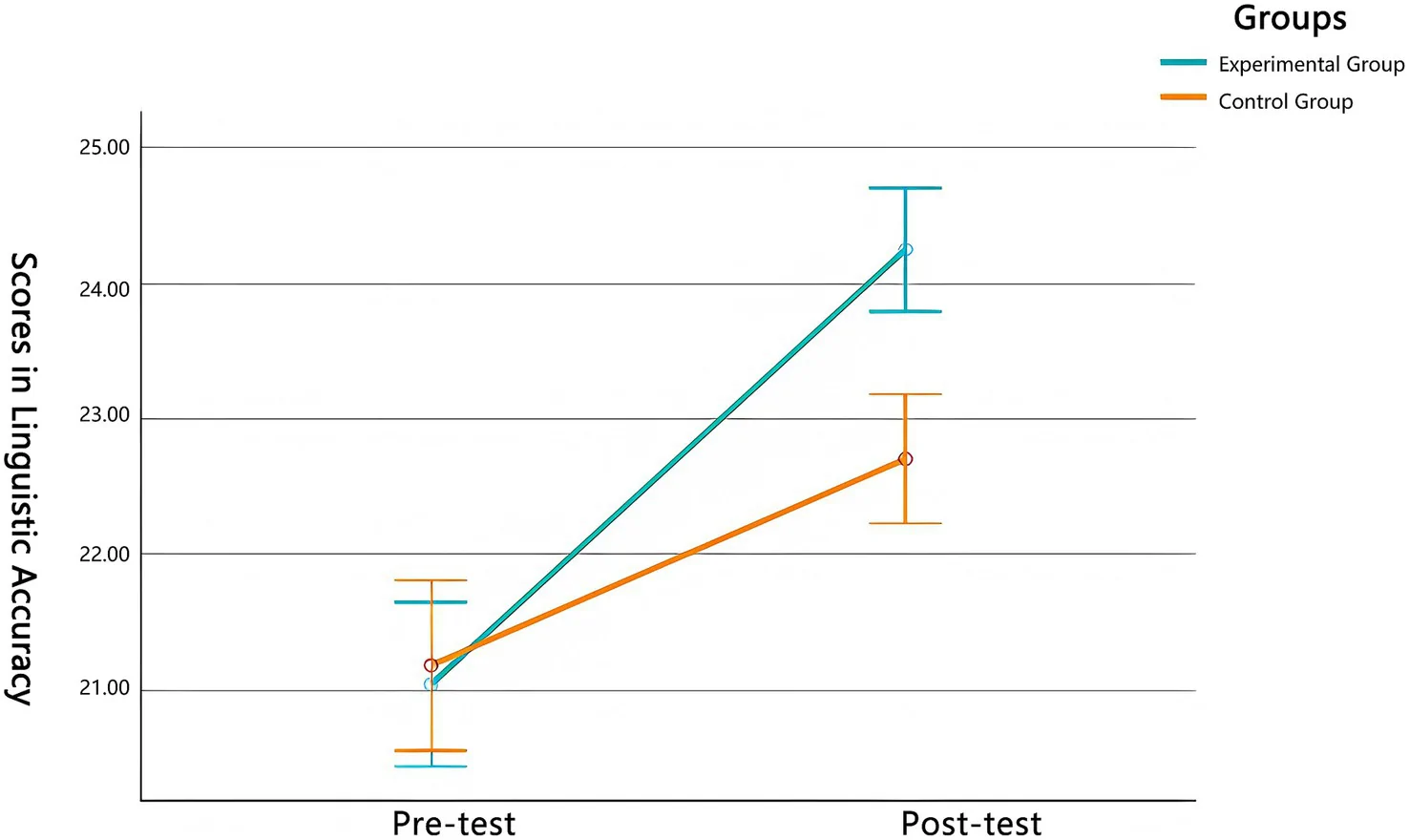
Interaction effect of time and group on linguistic accuracy scores in pre- and post-test interpreting. Error bars represent 95% confidence intervals.

**Figure 6:**
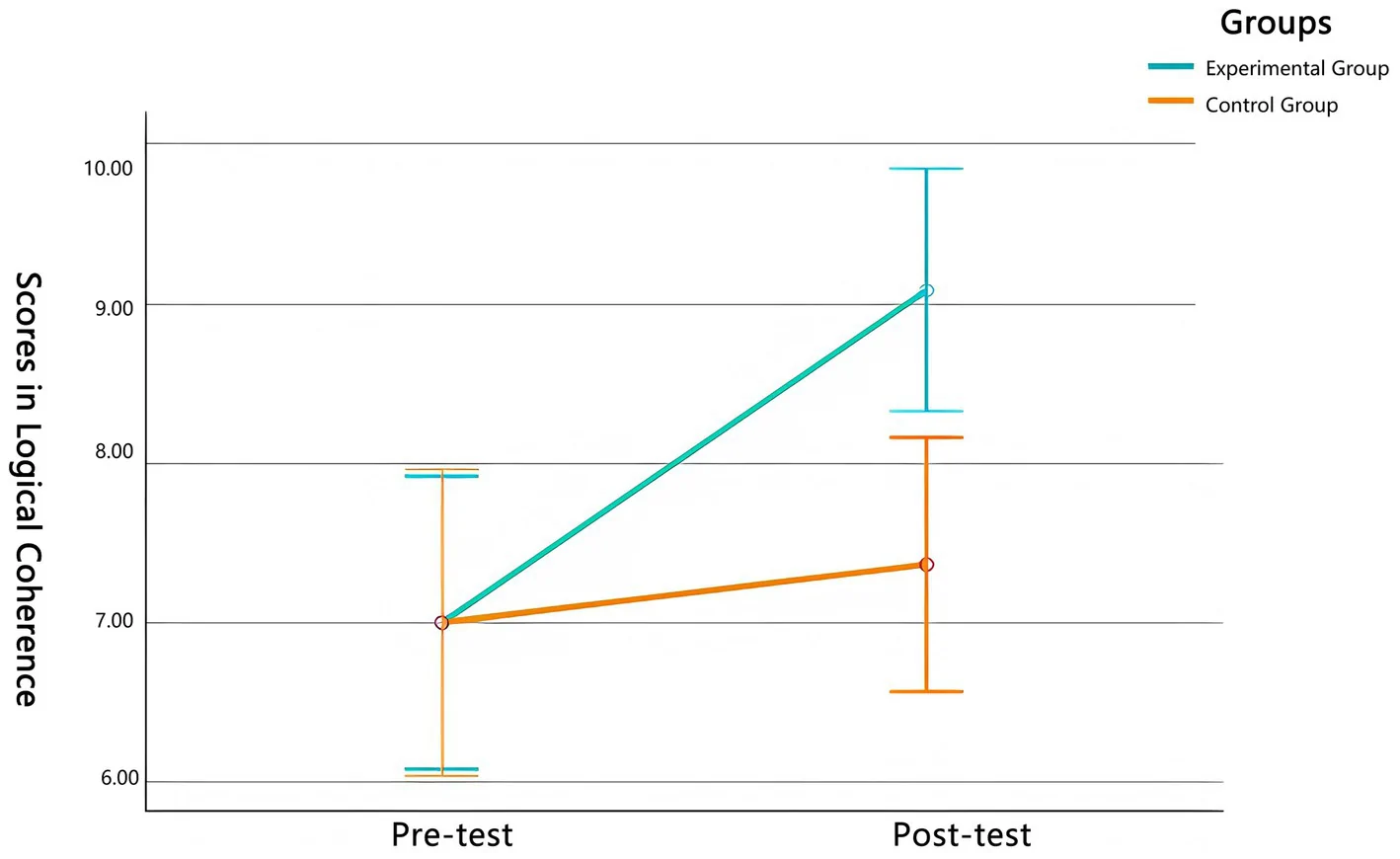
Interaction effect of time and group on logical coherence scores in pre- and post-test interpreting. Error bars represent 95% confidence intervals.

The overall between-group gain was 4.24 points (sum of sub-scores = 4.18, due to rounding). Accuracy contributed 1.69 points and coherence 1.72 (together 82%), fidelity 0.95 and fluency −0.18. Although not significant, the fidelity contribution draws attention because it arises from the control group’s decline rather than from any gain in the experimental group. Since four dimension-level ANOVAs were computed without correction for multiple comparisons, the coherence effect (*p* = 0.025) would not survive a Bonferroni or Holm adjustment. The dimension-level results are therefore framed as exploratory and are interpreted through effect sizes rather than significance thresholds. The two dimensions on which the intervention showed an advantage are also the two on which the system’s scores aligned most closely with human ratings in the pilot check (Section 3.5), whereas fidelity was weak on both counts. This correspondence is examined in Section 5.1.2.

### 4.2 SRL profile and its relation to performance gains

#### 4.2.1 Reliability, structural validity, and distribution

Internal consistency was high for the full scale (Cronbach’s *α* = 0.91, 95% CI [0.84, 0.95]) and for three subscales: Execution and Monitoring (0.85), Evaluation and Reflection (0.81), and Emotional Motivation (0.81), all exceeding the 0.800 benchmark. Pre-learning Planning reached only 0.61, below the conventional 0.70 threshold. As stated in Section 3.3.4, it is still retained for theoretical completeness, which is not unusual for a short subscale in exploratory research (Hair et al., 2010). But all findings concerning it are interpreted with corresponding caution.

Structural validity was supported by item-total and inter-dimension correlations (Table 4). Each dimension correlated strongly with the total scale (*r* = 0.67–0.89, all *p* < 0.001). Inter-dimension correlations were moderate (*r* = 0.34–0.66), with the association between Pre-learning Planning and Emotional Motivation being the only non-significant one. These four dimensions are therefore related but not redundant. Since *N* = 24 precluded formal factor analysis (Costello and Osborne, 2005), this evidence is preliminary, and the four-dimension structure is best regarded as provisional.

**Table 4:** Correlation matrix between dimensions and the total scale.

Dimensions	Pre-learning planning	Execution and monitoring	Evaluation and reflection	Emotional motivation	Total scale
Pre-learning planning	1				
Execution and monitoring	0.46^*^	1			
Evaluation and reflection	0.57^**^	0.66^***^	1		
Emotional motivation	0.34	0.48^*^	0.49^*^	1	
Total scale	0.67^***^	0.86^***^	0.89^***^	0.70^***^	1

*N* = 24. *p < 0.05, **p < 0.01, ***p < 0.001.

As four subscales comprise different numbers of differently worded items, their means are not directly comparable. The comparisons below are therefore read as relative patterns of self-reported engagement rather than absolute differences in how much students self-regulated. Execution and Monitoring (*M* = 3.92, *SD* = 0.65) and Evaluation and Reflection (*M* = 3.87, *SD* = 0.57) approached 4.0, while Emotional Motivation (*M* = 3.06, *SD* = 0.63) and Pre-learning Planning (*M* = 3.01, *SD* = 0.54) hovered around the scale midpoint (Table 5). Dispersion was widest for Execution and Monitoring and narrowest for Pre-learning Planning.

**Table 5:** Descriptive statistics and internal consistency reliability of the SRL questionnaire (experimental group).

Dimension	Item	Cronbach’s α	95% CI	Mean	SD
Pre-learning planning	1, 2, 3	0.61	[0.23, 0.82]	3.01	0.54
Execution and monitoring	4, 5, 6, 7, 8, 9,	0.85	[0.74, 0.93]	3.92	0.65
Evaluation and reflection	10, 11, 12, 13, 14, 15, 16	0.81	[0.67, 0.91]	3.87	0.57
Emotional motivation	17, 18, 19, 20	0.81	[0.65, 0.91]	3.06	0.63
Overall SRL	1–20	0.91	[0.84, 0.95]	3.60	0.49

*N* = 24 (Experimental Group). CI = Confidence Interval. Scored on a 5-point Likert scale (1 = never/strongly disagree, 5 = always/strongly agree).

#### 4.2.2 Correlation between SRL and interpreting performance gains

Pearson correlation analysis was conducted, linking the four SRL dimensions and interpreting score gains. Shapiro–Wilk tests confirmed normality for all variables (*p*s > 0.05). Only Execution and Monitoring correlated significantly with score gains (*r* = 0.42, *p* = 0.041, 95% CI [0.02, 0.70]). The remaining three did not (Table 6).

**Table 6:** Pearson correlation analysis between SRL dimensions and interpreting score gains (experimental group).

SRL dimension	*r*	95% CI	*p*
Pre-learning planning	0.29	[−0.13, 0.62]	0.170
Execution and monitoring	0.42*	[0.02, 0.70]	0.041
Evaluation and reflection	0.10	[−0.32, 0.48]	0.653
Emotional motivation	0.14	[−0.28, 0.51]	0.516

*N* = 24. *p < 0.05.

A multiple linear regression was performed to examine each dimension’s unique contribution (Table 7). Multicollinearity was acceptable (VIFs ≤2.20). The model accounted for 28.3% of the variance in score gains, though it did not reach significance overall [*F*(4, 19) = 1.87, *p* = 0.157]. Execution and Monitoring remained the only statistically significant predictor of score gains (*B* = 2.51, 95% CI [0.14, 4.88], *β* = 0.591, *p* = 0.039). Pre-learning Planning, Evaluation and Reflection, and Emotional Motivation did not contribute uniquely. With *N* = 24, the model is underpowered, and the coefficients are reported here as descriptive complements to the bivariate correlations rather than as independent confirmation of unique effects.

**Table 7:** Multiple regression analysis predicting interpreting score gains.

Predictor	*B*	SE	*β*	*p*	95% CI
(Constant)	0.01	4.04		0.998	[−8.44, 8.46]
Pre-learning planning	1.40	1.22	0.273	0.267	[−1.16, 3.95]
Execution and monitoring	2.51	1.13	0.591	0.039	[0.14, 4.88]
Evaluation and reflection	−2.14	1.41	−0.437	0.146	[−5.08, 0.81]
Emotional motivation	−0.09	1.01	−0.020	0.931	[−2.19, 2.02]

*N* = 24. *R*^2^ = 0.283, *F*(4, 19) = 1.87, *p* = 0.157. CI = confidence interval; SE = standard error.

### 4.3 Students’ engagement with the automated feedback

To understand the behaviors and perceptions underlying the patterns above, semi-structured interviews with seven students from the experimental group (two high-, three mid-, and two low-scoring) were analyzed and coded in NVivo following Braun and Clarke’s (2006, 2019) reflexive approach to thematic analysis. Coding proceeded through several passes, yielding 69 codes and 262 coded references. These were organized under the four *a priori* SRL dimensions and consolidated into 16 categories; the full code list is retained in Appendix D. Consistent with reflexive TA, theme labels continued to be refined during analysis and writing rather than fixed in advance. Given the size of the sub-sample, the analysis illustrates and helps explain the quantitative patterns rather than establishing how widespread any view is (Section 3.3.5). Table 8 sets out the coding framework under the four SRL dimensions, with coding frequencies and representative quotations.

**Table 8:** Interview coding framework and coding frequencies (*N* = 7).

SRL dimension (refs)	Categories	Code	*n*	Refs	Representative quotation
1. Pre-learning planning (45)	1.1 Goal orientations	Feedback-driven target setting / target setting	5	8	“Last week my fluency was only 21, so this time I told myself not to freeze up and to cut down on the ‘ums’.” (S3)
		Outcome-focused perception	1	1	“Set a goal? What kind of goal? You mean, how many points to get?” (S2)
		Protective goal setting	2	2	“I do not dare set the target too high, in case I fall short and feel even worse.” (S3)
		Material orientation / topic orientation	6	8	“Before each interpreting practice, I go through the textbook, run over the vocabulary, underline the proper nouns…” (S3)
	1.4 Planning impediments	Inconsistent planning	4	5	“I basically just open the app and start.” (S7)
		Time allocation constraint / fragmented practice planning	3	3	“There are just too many classes, plus assignments and presentations; I can only squeeze interpreting practice into whatever gaps I find.” (S4)
2. Execution and monitoring (68)	2.1 Attentional regulation	Feedback-driven focus / selective attention	4	10	“My attention is more focused, and it’s not so aimless.” (S5); “Knowing that my fidelity was previously low, I would remind myself not to miss any information.” (S6)
	2.2 Strategic adaptation	Remedial strategy execution	4	4	“If it’s a vocabulary problem, I look it up and take notes; if it’s a logic problem, I re-analyze the source text structure; if it’s a fluency problem, I practice in front of a mirror.” (S1)
	2.3 Effort calibration	Cognitive overload	6	8	“It’s like four little people fighting in my head.” (S1); “I cannot really spare my attention to monitoring myself.” (S4)
		Increased practice / increased effort investment	4	12	“Most of the time the scores aren’t very good, so I end up practicing more than before… but practicing more really works.”(S5)
		Effort regulation (practice frequency unchanged)	2	2	“The frequency is about the same.” (S6)
		External time pressure	3	3	“There really is not that much time, and sometimes several assignments have to be submitted on the same day.” (S7)
	2.4 Real-time awareness	System-triggered monitoring	4	4	“When practicing, I definitely check the scores and decide whether to practice a few more times based on them.” (S5)
		Unsystematic monitoring	3	3	“In the past, practicing interpreting felt really aimless, and after finishing I had no idea how well I performed.” (S2)
3. Evaluation and Reflection (86)	3.1 Diagnostic analytics	Diagnostic signal / problem diagnosis	7	14	“In the past, teachers graded homework and we might only see the final results, but the system breaks down the problems.”(S6)
		Blind spot identification	3	5	“When I saw the transcript of my own filler words for the first time, I was really shocked.” (S2)
	3.2 Feedback processing	Instant correction	6	8	“In the past we would submit on Tuesday, but the teacher would not discuss it until Friday’s class… now I see the feedback immediately after finishing, so the impression is much deeper.” (S4)
		Actionable feedback preference/feedback use	6	9	“The reference is actually more useful than the scores. The scores only tell me that I did not do well, but the reference shows me how I should interpret it.” (S1)
		Black-box feedback	5	5	“I used logical connectors, but it gave me a 0.” (S4)
	3.3 Evaluative comparison	Overall score focus / temporal comparison	6	12	“I look at the overall score first, as it’s the most straightforward.” (S1); “I see if the overall score went up or down compared to last time.” (S2)
		Selective inspection	7	10	“I just give them a quick glance.” (S3); “I do not look at the transcripts and annotations every time.”(S7)
	3.4 Meta-cognitive integration	Unsystematic reflection	4	5	“I only look at the score without really digging into the reasons behind it.” (S7)
4. Emotional motivation (63)	4.1 Affective regulation	Evaluative anxiety	4	4	“Now every submission feels like an exam with instant results.” (S1); “It always feels like someone is watching you.” (S7)
		Anxiety reduction	3	4	“There’s still pressure sometimes, but not that old feeling of having no idea where I stood… the score is not my final grade.”(S5)
		Emotion regulation	6	6	Deferral: “I close the page first and deal with it later.” (S3); habituation: “I gradually got used to it and stopped caring so much about the score.” (S4)
		Shame avoidance	3	4	“The score was just too ugly and I could not stand it.” (S1); “I know I did not do well, so I do not want to look at it. It’s embarrassing.” (S7)
	4.2 Self-efficacy dynamics	Fluctuating self-efficacy / low self-efficacy	4	6	“When the score is high I feel okay; when it’s low I doubt myself.” (S1); “The reference is so good, there’s no way I’ll ever reach that level.” (S3)
		Increased agency	4	4	“How many times I do it, when I do it, how I do it—that’s all under my control.” (S4)
	4.3 Motivational drivers	Performance-driven motivation	4	7	“My initiative has definitely gone up, but not because ‘I love interpreting’; it’s because ‘the score looks bad, so I need to practice again’.” (S1)
	4.4 Technological orientations	Tool neutrality	1	2	“Just having a tool does not mean I’ll practice more.” (S3)

*n* = number of interviewees (of 7) contributing at least one reference; Refs = number of coded references. Dimension totals in the first column cover all sub-themes in that dimension; the table reports only the sub-themes discussed in Sections 4.3 and 5. The full three-level framework (69 codes, 16 categories) are given in Appendix D. Where two closely related sub-themes are reported on one line (e.g., Material Orientation / Topic Orientation), n and Refs are the union of the two nodes. Quotations are translated from Chinese and lightly edited for readability; ellipses mark omitted material. Given the sub-sample size they carry no claim about prevalence in the wider population.

Evaluation and Reflection and Execution and Monitoring were the two most densely coded dimensions (86 and 68 references respectively), followed by Emotional Motivation (63) and Pre-learning Planning (45). Coding density partly reflects the interview protocol and is not read here as an index of importance. The discussion below therefore begins with Execution and Monitoring, the only dimension that predicted score gains.

Within Execution and Monitoring, 18 references from six of the seven students described the score as the trigger for concrete action (System-triggered monitoring, 4 refs; Feedback-driven focus and selective attention, 10; Remedial strategy execution, 4). Students reported that practice had previously been “aimless” (S2) and that they now decide “whether to practice a few more times” on the basis of the score (S5), with remedial routines differentiated by problem type (S1). Four students (S1, S2, S5, S7) reported practicing more than before, whereas S3 and S6 reported that their practice frequency was essentially unchanged (“just having a tool does not mean I’ll practice more’, S3; “the frequency is about the same,” S6). Eight references from six students fell under Cognitive Overload. Monitoring four dimensions online was described as “four little people fighting in my head” (S1) and, as something students “cannot spare the attention for” (S4). Self-monitoring was thus frequent but behavioral rather than concurrent and strategic.

Evaluation and Reflection combined high engagement with shallow processing. The total score was checked first by six of seven students (Overall Score Focus), and the sub-scores and transcript did surface previously invisible problems (Diagnostic Signal and Problem Diagnosis, 14 references from all seven students; Blind-spot Identification, 5 references from three students). Inspection was nonetheless superficial for most students. All seven were coded under Selective Inspection, describing sub-scores that were only given “a glanced” (S3) or transcripts and annotations not consulted “every time” (S7), and five references from four students under Unsystematic Reflection indicate that causal analysis was rare. Five students described the system as opaque (Black-box Feedback), and six preferred the reference translation to the scores because it specified a remedy rather than a deficit (Actionable-feedback Preference/Feedback Use, 9 references).

Pre-learning Planning was the least coded dimension. Eight references from five students cited competing coursework and unstable routines (Inconsistent Planning, 5; Time Allocation Constraint, 2; Fragmented Practice Planning, 1); a further three references from three students under External Time Pressure were coded within Execution and Monitoring. Where goals were set, they were derived from the previous session’s score rather than from analysis of the upcoming task (S3), were sometimes equated with the score itself (S2), or were deliberately kept low (S3). Anticipatory preparation was mostly oriented to the textbook and topic rather than to the system (all but S4; 8 references).

Emotional Motivation was internally divided. Seven references coded as Performance-Driven Motivation separated effort from interest (“not because ‘I love interpreting’… it’s because ‘the score looks bad so I need to practice again’,” S1). Anxiety moved in both directions. Two students (S1, S7) felt more exposed, while three felt less pressure than under teacher-collected submission (S2, S5, S6). Three students deferred engagement after a low score (S1, S3, S5), one reported habituating and disengaging (S4), and one described little emotional reaction either way (S6). S7 combined heightened evaluative anxiety with avoidance of poor performances (“it’s embarrassing”), a shame-avoidance pattern examined in Section 5.3.3. Self-efficacy was described as score-dependent and unstable (S1), and the reference, though the most actionable output, was also demoralizing for one student (S3).

## 5 Discussion

This study posed three research questions, and the results reported in Section 4 yielded three corresponding findings. Practice with the automated scoring system led to significantly greater improvement than conventional practice, but only on dimensions related to linguistic form and structure. Gains in information fidelity and delivery fluency were no greater than those of the control group. Students’ self-regulatory profile was also uneven. It was comparatively strong during and immediately after practice, but much weaker in the planning phase, and only the former was associated with performance gains. The interviews help explain this discrepancy. Students consistently measured their performance against the score, but rarely explained it to themselves, hindered by real-time cognitive load, by the opacity of the scoring algorithm, and by the need to protect themselves from a low score. Read through Zimmerman’s (2000, 2002) cyclical model, these are not three findings but one. The intervention reinforced the performance phase, reached self-reflection only in its lower-effort form, and left forethought largely untouched. Given the quasi-experimental design and the small questionnaire and interview sub-samples, the accounts developed below should be read as interpretations consistent with the data rather than as demonstrated mechanisms (see Section 6.3).

### 5.1 Effectiveness of automated scoring and its uneven distribution (RQ1)

#### 5.1.1 Overall effectiveness

The experimental group outperformed the control group at post-test with a large effect size (Cohen’s *d* = 1.03, 95% CI [0.41, 1.64]), and the time × group interaction on total scores was large (*η*^2^ₚ = 0.290). Feedback functions as a pillar of students’ SRL and as one of the most powerful influences on learning and achievement (Hattie and Timperley, 2007). However, its effects are far from uniform. A recent meta-analysis of 435 studies found only a medium overall effect (*d* = 0.48) with substantial heterogeneity, indicating that what matters is what kind of feedback is delivered and what it targets (Wisniewski et al., 2020). The present effect is above that average, which raises the question of what the automated condition supplied that conventional practice did not.

Immediacy and individualization, which are two of the automated scoring system features, might help explain this. Even though they are intertwined in the experiment, they are discussed separately for clarity. Immediacy could help narrow the window between error and correction. Interpreting is a high-load operation requiring simultaneous management of listening and analysis, memory, production, and coordination (Gile, 2009). Under such cognitive load, specific details, such as word choice, fillers, mistranslations, or omissions, decay from memory quickly. The automated scoring system delivers scores and transcripts immediately once a task is completed, while teacher feedback is often delayed by several days, by which point the memory of the original performance has faded (Shute, 2008). S4 described this explicitly: “In the past, we would submit assignments on Tuesday, but the teacher would not discuss them until Friday’s class. By then, I could not even remember how I had interpreted it... now, I see the feedback immediately after finishing, so the impression is much deeper.”

Individualization might also help close the gap. According to Shute’s (2008) taxonomy, the platform delivers multi-dimensional knowledge of results and knowledge of the correct response, but not elaborated feedback explaining why an error occurred. Therefore, what distinguishes it from the control group’s collective review is not the depth of explanation but that every student obtains a comparison against their own output, something whole-class review cannot provide for each student.

These are plausible accounts rather than demonstrated mechanisms. The design was quasi-experimental with intact classes, so class-level differences such as peer dynamics cannot be excluded, and the two features above were confounded by design. Another possibility is increased time on task, as students reported practicing more once they got the scores (“Most of the time the scores aren’t very good, so I end up practicing more than before…but practicing more really works,” S5). However, this was not uniform. Four of the seven interviewees reported practicing more, but S3 and S6 reported unchanged frequency (“just having a tool does not mean I’ll practice more,” S3), so increased effort accounts for part of the group-level gain. Rather than a rival explanation, increased practice is best understood as part of the intervention’s effect, because it was prompted by the scores. Still, this means that the improvements cannot be explained purely by the information in the feedback.

#### 5.1.2 An uneven effect across dimensions

The dimensional asymmetry reported in Section 4.1.2 becomes sharper when each group’s gain is calculated against the points available at pre-test. The experimental group consumed 81.1% of its remaining headroom on linguistic accuracy (21.04 → 24.25 of 25) and 69.3% on logical coherence (7.00 → 9.08 of 10), but only 4.4% on delivery fluency and 0.5% on information fidelity. Since the coherence interaction is exploratory and would not survive correction, the argument below rests on this distribution of movement. The coherence effect is treated as provisional throughout, supported by its effect size (Cohen’s *d* = 0.93, 95% CI [0.31, 1.53]) rather than by its *p*-value.

There are two explanations which should be kept apart here. The first is a genuine ceiling. There is too little headroom at pre-test for improvement. The second is restricted range and scale insensitivity. While there is headroom in principle, the pre-test variance is quite narrow, and within that tight band, the scale is too coarse to reliably capture meaningful change. Neither of these applies to fidelity, which had the largest absolute headroom of the four dimensions (8.87 points) and the lowest proportional pre-test score (74.7% of maximum), yet moved 0.04 points. Fluency, by contrast, is a case of the second. With 4.10 points still available, the pre-test SD was only 1.85 (CV = 7.1%, the narrowest in the battery). The control group’s slightly larger gain (+0.36 vs. +0.18) is well within measurement noise on such a coarse scale. The null shows less about the skill than about the instrument’s ability to detect movement in the range where these students actually varied.

What the two responsive dimensions have in common is not difficulty, but how easily they can be broken down and diagnosed. A mistranslated term or a missing connective can be time-stamped, named, and corrected without reorganizing the underlying process. This places feedback at the task-detail level, where it is most reliably actionable (Kluger and DeNisi, 1996) and where noticing operates (Schmidt, 1990). It also parallels the salience asymmetry found in corrective-feedback research, where explicit feedback benefits rule-governed targets but not diffuse ones (Shintani et al., 2014). Accuracy now stands at 97.0% of maximum, so this gain is near saturation and cannot be taken further.

Fidelity stands out as one true null result, and two features of the construct point to that direction. The problem of fidelity originates earlier, in comprehension and note-taking, so feedback on the finished rendition only flagged a symptom, not addressing the cause which has already gone. For the same reason, fidelity resists automated modelling. In the pilot, it correlated at only *r* = 0.12 (ns), compared with 0.79 and 0.62 for coherence and accuracy (Table 1). Diffuseness undercuts actionability and credibility at the same time.

So feedback only produces a gain when the problem can be broken into points the learner can act on, when the signal is reliable enough to be trusted, and when the scale is sensitive enough to capture change. Accuracy and coherence met all three conditions; fidelity failed the first two, fluency the third. The practical implication is limited. What improved was surface linguistic form, carrying 35 of the rubric’s 100 points, while the 65 points measuring meaning transfer and delivery were untouched. So addressing those problems means moving feedback earlier (on note-taking and comprehension) and making the scales sensitive enough to detect real differences.

### 5.2 An unbalanced SRL profile and its relation to performance gains (RQ2)

The questionnaire data (Section 4.2) describe an SRL profile that is comparatively strong during and after the task but weak before it. More importantly, the profile does not correspond to actual performance (Section 4.2.2). Only Execution and Monitoring correlated significantly with score gains (*r* = 0.42, 95% CI [0.02, 0.70]), while the second-highest-scoring dimension, Evaluation and Reflection, showed a near-zero association (*r* = 0.10). Read through Zimmerman’s (2000, 2002) three-phase cycle, these are not four independent findings but one structurally uneven reactivation of the cycle. The profile observed after the intervention is strongest in the performance phase, partial in self-reflection, and weakest in forethought and the motivational beliefs.

Execution and Monitoring was the sole significant predictor in the regression model (*B* = 2.51, 95% CI [0.14, 4.88], *β* = 0.591, *p* = 0.039; *R^2^* = 0.283), suggesting that the bivariate association is not simply an artifact of shared variance. Since the overall test was not significant, this coefficient should be interpreted descriptively. The result is consistent with deliberate practice theory (Ericsson, 2000). The system enables a tight production–diagnosis–reproduction loop, and students described concrete behavioral compensation, moving from a single attempt to “at least twice” (S1) or “a few more times” (S5). The interview data, however, revealed a more nuanced process. Monitoring took a behavioral form (repeating the same material until the score improved) rather than the strategic adjustment Zimmerman’s model predicts. Students explicitly attributed this to the lack of spare capacity during production (S1, S4, S6, S7). When comprehension, memory and production compete for limited processing capacity, resources for online metacognitive control are the first to be sacrificed (Gile, 2009; Seeber, 2011). Self-monitoring, then, was reduced to the most manageable form.

The remaining three dimensions did not translate into gains. Despite carrying the second-highest mean, Evaluation and Reflection showed a near-zero raw correlation and, in the regression, a coefficient that reversed sign and grew in magnitude (*B* = –2.14, 95% CI [−5.08, 0.81], *β* = −0.437, *p* = 0.146). Table 4 shows it correlates strongly with Execution and Monitoring (*r* = 0.66, *p* < 0.001), the collinearity classically associated with statistical suppression. Once behavioral engagement is held constant, self-reported reflection contributes no unique positive variance to gains and may even compete with it for students’ limited time. Given the sample size and the non-significance of the coefficient, this is one plausible statistical account rather than a demonstrated mechanism. What it does establish is that high self-reported reflection cannot be assumed to indicate productive reflection. Pre-learning Planning and Emotional Motivation both hovered near the midpoint and were unrelated to gains. These two dimensions were weak together rather than independently. Zimmerman locates self-motivation beliefs within the forethought phase, so if forethought is skipped, the point in the cycle at which those beliefs are normally formed is skipped with it. Section 5.3 provides the interview-based account of why these dimensions failed to convert engagement into improvement.

### 5.3 A fractured cycle of feedback use (RQ3)

#### 5.3.1 Rich diagnostics, selective use of feedback

Zimmerman (2000) separates two distinct self-reflection sub-processes: self-judgment (comparing performance against a standard) and causal attribution (analyzing why errors occurred). Table 8 shows self-judgment operating at very high frequency. Almost every student reported checking the overall score first and then comparing it against previous attempts (S1, S2, S3, S5, S6, S7). Causal attribution was reported far less consistently. S7 looked at the score “without really digging into the reasons behind it,” and S2 “rarely do[es] a detailed analysis.” This was not because diagnostic material was not available, since alongside the score, the platform provided a transcript, pronunciation annotations, sub-scores for each dimension, and a reference translation. Some students used these efficiently. S1 found the reference more useful than the score because it showed how to interpret the passage. The gap is that the materials were used selectively rather than an integral part of the feedback event (S1, S3, S6, S7). A plausible interpretation is that self-judgment is less effortful than causal attribution, and is therefore the sub-process students default to under time pressure.

Opacity compounded the problem. Several students described the scoring as unpredictable: “I do not know how it calculates the score, so I just let it be” (S2); “I used logical connectors, but it gave me a 0” (S4). Butler and Winne’s (1995) claim that feedback is productive only when it triggers active regulation therefore needs a qualification here. The materials capable of triggering that regulation existed, but an opaque scoring layer discouraged the very engagement needed to use them. This is also the barrier that explainable-AI approaches are meant to address (Jiang and Zhang, 2025).

#### 5.3.2 Backward-looking planning

Pre-learning Planning returned the lowest mean of the four dimensions with no significant association with score gains. Read alone, this invites the conclusion that students simply did not plan. The interviews suggest something different. Planning did not so much disappear as change direction.

Where students did set goals, those goals derived mostly from the previous session’s score rather than from any analysis of the upcoming task. S3 set out to beat the fluency score of 21 recorded the week before. S4 reported that an earlier low score “stays on my mind.” S6 “usually just hope[d] to do a bit better than last time — fewer pauses, keep a bit more of the information.” In Zimmerman’s (2000, 2002) terms, these are recognizable acts of goal setting, but their content originates in the previous self-reflection phase. The forethought phase has not been eliminated, but it has been absorbed backward into the previous reflection–execution loop. It retains the form of goal setting, but loses its function as an independent, forward-looking analysis of task demands.

Two further features support this reading. First, the goal and the score had become difficult to distinguish. Asked whether they set goals before practicing, S2 responded with a question: “Set a goal? What kind of goal? You mean, how many points to get?” The score had become the learning goal itself. In addition, several students engaged in protective goal setting, adjusting targets downward to limit the affective cost of failure. For instance, S3 “did not dare” set a high target, and S6 avoided setting goals that involved major improvement. Goal setting is driven by a score already received, and shaped by anticipated disappointment at the next one. This makes it structurally incapable of performing the anticipatory work that Zimmerman’s model assigns to the forethought phase. Moreover, the anticipatory planning that did survive was not oriented toward the system but to the syllabus, such as reviewing textbook glossaries and proper nouns (S3, S5), or predicting difficulties based on the topic (S1). The capacity for forethought was present. What the automated scoring condition supplied was a lower-effort, retrospective substitute for it. A packed timetable made anticipatory preparation more expensive. S4 could “only squeeze interpreting practice into whatever gaps I find,” while S7 “basically just open[ed] the app and start[ed].”

#### 5.3.3 The affective divergence

The questionnaire showed Emotional Motivation near the scale midpoint (*M* = 3.06) with no significant relationship to score gains (*r* = 0.14, 95% CI [−0.28, 0.51], *p* = 0.516). The interviews indicate that this null result reflects not affective indifference but affective divergence.

Anxiety moved in both directions. Two students reported an increase, both attributing it to the loss of collective anonymity. S1 felt that “now every submission feels like an exam.” S7 contrasted the “sense of security in blending into the crowd” under collective submission with the feeling that “someone is watching you” on the platform, and remained worried that scores might be used as a reference despite the teacher’s assurance. Three students reported the reverse. For S2, the relevant contrast was with teacher scrutiny rather than peer visibility. S2 had previously submitted at the deadline to avoid close inspection, whereas on the platform “that does not really matter.” For S5, the reduction came from less uncertainty (“not that old feeling of having no idea where I stood”), and S6 was just simply “not as nervous using the platform.” Automated scoring thus simultaneously depersonalizes the evaluator and makes the record permanent and individually visible. Which effect dominates depends on which audience a student regards as evaluative. That is a distinction that an aggregate subscale mean conceals.

A second pattern concerns how students managed negative affect once a low score arrived. Three of the seven described deliberate deferral, setting the passage aside for half a day or longer before returning to it (S1, S3, S5). This is adaptive emotion regulation in Zimmerman’s (2000, 2002) self-reaction terms, and for S5 it was precisely what made productive re-engagement possible. However, it also erodes the immediacy mechanism in Section 5.1.1. The temporal advantage was partly surrendered by the students who most needed the diagnostic information, and surrendered for affective rather than cognitive reasons. S4 described a less adaptive endpoint of the same process, and “gradually got used to it and stopped caring so much about the score.”

Finally, where the system did drive effort, the driver was frequently shame avoidance rather than learning motivation. Shame operated in both directions. It motivated practice. S1 could not stand a score that was “too ugly,” and S4 did not want a record that was “a total mess.” But the same emotion also triggered avoidance. S7 declined to look at poor performance because “it’s embarrassing.” This bi-directionality offers an explanation for the null correlation. Emotional Motivation was not inert but internally opposed, with approach and avoidance responses to the same affective trigger cancelling one another in aggregate. It also qualifies the resubmission function, which S7 described in frankly instrumental terms: “you can record several times to push the score up.”

Self-Determination Theory (Ryan and Deci, 2000) helps specify which needs were implicated. Autonomy was enhanced. For instance, S4 noted that “how many times I do it, when I do it, how I do it … that’s all under my control.” On the other hand, competence was often undermined, and not only by low scores. S3 found the reference “so good, there’s no way I’ll ever reach that level.” So the resource that Section 5.3.1 identified as the most actionable output also served as a discouraging reminder of how far students still had to go. S6 put it bluntly that “what I cannot do I still cannot do.” Relatedness was weakened when submissions shifted from collective to individually visible, with no human response yet arrived. When both competence and relatedness are unmet at the moment of self-reaction, motivation tends to reorganize around performance rather than mastery goals, which is precisely the orientation dominant here: “my initiative has gone up, but not because ‘I love interpreting and want to practice every day’. It’s because ‘the score looks bad so I need to practice again’.”(S1).

#### 5.3.4 Toward synchronized hybrid feedback

These findings imply a more targeted recommendation than simply combining automated and teacher feedback. The two are already combined in this design, just not synchronized. Automated scoring’s comparative advantage lies in the performance phase. Its immediacy, repeatability, and quantification sustain the deliberate-practice loop that drove score gains, and no redesign should sacrifice that. The gap is not the absence of teacher feedback but its distance, in time and in individuation, from the automated scoring and feedback. The synchronization issue is not just informational. It is also affective, because by the time students receive a human explanation of why, their self-reaction has already formed. In some cases, it has already been set aside or faded. Research on interpreter feedback supports this interpretation. Both trainers and trainees regard feedback as most useful when it is specific to the individual performance (Domínguez Araújo, 2019). Peer-assessment finding also align with this view, showing that feedback delivered near the performance and focused on recognizable individual errors encourages reflection rather than defensive self-protection (Yang et al., 2025). That study did not manipulate timing or individuation, so the parallel is suggestive rather than conclusive. Nonetheless, it points to the likelihood that teacher feedback can be more effective if it moves closer in time and in individuation. For instance, brief individualized comments could be delivered before the following week’s collective review, so that a human explanation reaches the student while the automated diagnostics are still fresh in mind. Alternatively, they could design short forward-looking task-analysis exercise before each new assignment, anticipating difficulties rather than recalling last week’s score, so as to restore an independent forethought phase.

## 6 Conclusion

### 6.1 Summary of findings

Drawing on experimental, questionnaire, and interview data, this study examined how automated interpreting scoring reshapes students’ interpreting performance and their SRL process. It addresses the three questions in the Introduction: how such systems function once embedded in real classroom practice, which phases of the SRL cycle they reach, and how students use automated feedback.

In response to RQ1, the use of an automated scoring system was associated with measurable gains in overall interpreting performance (*d* = 1.03, 95% CI [0.41, 1.64]), but these gains were confined to linguistic form. Linguistic accuracy and logical coherence improved substantially, but information fidelity and delivery fluency did not. Gains appeared only where the deficit was decomposable, the signal reliable, and the scale sensitive. Fidelity failed the first two conditions and fluency the third. The yield is therefore bounded to surface linguistic form.

In response to RQ2, students in the experimental group exhibited an uneven SRL profile, comparatively strong during and immediately after practice but weak before it, with Pre-learning Planning and Emotional Motivation near the scale midpoint. Only Execution and Monitoring was associated with score gains, and it was the sole significant predictor in the regression model. Evaluation and Reflection, despite carrying the nominally second-highest mean, showed a near-zero association, so high self-reported reflection cannot be equated with productive reflection. The post-intervention profile is strongest in the performance phase, partial in self-reflection, and weakest in forethought and the motivational beliefs.

In response to RQ3, the interviews explain how the cycle fractured. Self-judgment was near-universal, with students checking the score and comparing it against previous attempts, but causal attribution was reported far less consistently. There are three constraints: the lack of spare capacity for real-time monitoring under interpreting load, the opacity of the scoring algorithm, and the affective cost of examining a poor performance. Planning did not disappear but reversed direction, deriving from the previous session’s score rather than from analysis of the upcoming task, and was frequently set defensively low. Emotional responses diverged rather than being absent. The same score prompts renewed practice in some students and avoidance in others, which accounts for the null correlation of Emotional Motivation.

### 6.2 Implications

These findings carry several implications. Theoretically, the findings refine two accounts. For Zimmerman’s cyclical model, they show that a feedback intervention can enter the cycle phase selectively. The performance phase is reinforced, but forethought is not only weak but structurally displaced. It is absorbed backward into the previous reflection phase as a retrospective substitute which has the form of goal setting but loses its anticipatory function. For feedback intervention theory, they specify three conditions under which task-level feedback converts into gains: decomposability of the problem, reliability of the signal, and sensitivity of the scale. They also indicate that failure on any one of them is sufficient to produce a null.

Pedagogically, the findings contribute to the improvement of hybrid feedback. In this study, automated and teacher feedback were combined but not synchronized. The comparative advantage of automated scoring lies in its immediacy and repeatability and this should be preserved. What is missing is a human explanation of why an error occurred. Such feedback is most useful when the diagnostics are still fresh, hence calling for shortening the lag between automated scoring and teacher’s explanation. A brief task-analysis exercise may help restore an independent forethought phase.

### 6.3 Limitations

Several limitations qualify these conclusions. Institutional constraints required intact classes. Although conditions were allocated by coin flip, class-level confounds such as peer dynamics cannot be excluded, and the relationships reported are best read as associative rather than causal. The intervention was also delivered as a package with immediacy, individualization, and increased time on task co-varied by design so it also speaks to the package as a whole. In addition, the scoring system is a proprietary product with undisclosed computational formulations, and its agreement with human raters was limited. Therefore results should be interpreted with caution. The human rubric matched the system’s framework in order to align the two scoring methods and rule out pre-test bias, but it also means that the experimental group’s advantage may reflect rubric familiarity. An independent measure would be more conclusive. With a small sample size, the present findings are best treated as preliminary. The contribution of the study may lie less in estimating the magnitude of any effect than in documenting that the approach can be implemented in an authentic classroom setting, and in offering provisional dimensions and initial estimates that a larger, adequately powered study might build on.

### 6.4 Future research

Future research could employ factorial designs that separate immediacy from individualization and automated from human feedback, and should assess performances on a rubric independent of the automated scoring system. Researchers might also develop rubric scales with greater sensitivity. Another direction is to test whether explainable scoring outputs increase causal attribution rather than self-judgment alone. Replication with larger and more diverse samples (across institutions, language pairs, and proficiency levels) is a precondition for generalizing any of the patterns reported here.

## Data Availability

The original contributions presented in the study are included in the article/supplementary material, further inquiries can be directed to the corresponding author/s.
