# Supplementary material for: Impact of automated scoring on interpreting performance and self-regulated learning: evidence from a pedagogical experiment
Source: Front Psychol. 2026 Sep 14;17:1867463. doi: 10.3389/fpsyg.2026.1867463 (PMC13617063; doi:10.3389/fpsyg.2026.1867463)
Supplement: Supplementary file 1 [file Supplementary_file_1.pdf]

## Appendices

### Appendix A　Pre- and Post-test

**Note:** This appendix presents the transcript of the original Chinese speeches of the pre-test and post-test used in this study, alongside their English translations. The Chinese texts are the actual materials administered to participants, and the English translations are provided for readers' reference. In case of any discrepancy, the Chinese original shall prevail.

### Pre-test (1’52” /350 characters)

教育交流合作是对外关系的重要组成部分。近年来，我们与多国的教育交流合作日益丰富，涵盖了学龄前教育、基础教育、职业教育与高等教育各个领域。我们积极拓展合作渠道，推动教育事业的共同发展。

比如我们与英国启动了“数学教师交流项目”，互派中小学数学教师。老师们带去了先进的教学方法，也带回了英国同行的创新经验。在坦桑尼亚，我们开展了数学教师的专业发展项目，通过线上线下相结合的方式分享经验资源，提升了当地数学老师的专业素养和教学能力，影响了无数学生，推动了当地的教育转型。

这样的教育交流，不仅帮助当地提升了教学水平，更让两国人民拉近了距离。老师们在传授知识的同时，用真诚赢得了学生的信任。学生们不仅拓宽了视野，也感受到了异国友谊。这种在日常教学中建立的信任，为两国关系打下了扎实的民间基础，让友好往来更加长久。

Translation:

Educational exchange and cooperation are important part of foreign relations. In recent years, our exchanges with other countries have expanded across preschool, basic, vocational, and higher education. We have actively broadened cooperation channels to promote common development in education.

For example, our “Mathematics Teacher Exchange Program” with the UK has facilitated reciprocal visits of primary and secondary school teachers, who introduced advanced teaching approaches and returned with innovative strategies from British teachers. In Tanzania, a blended online-offline professional development program for math teachers enhanced local skills and capacity, benefiting countless students and driving local educational transformation.

Such exchanges improve teaching while bringing our peoples closer. Teachers earn students’ trust through sincerity, and students gain broader perspectives and cross-cultural friendships. This trust, built in daily classrooms, lays a solid grassroots foundation for bilateral relations and enduring friendship.

**Post-test (1’43” /340 characters)**

数字创新正在迅速改变我们的生活和工作方式。无论是人工智能、大数据，还是物联网、移动支付，每一项创新都在推动社会变革。数字创新不仅提升了效率，还提供了全新的解决方案，解决了许多传统方式无法应对的问题。

在教育领域，在线教育平台和虚拟课堂打破了地域和时间的限制，使更多人能够获得优质教育资源。教育机构通过大数据分析可以更好地了解学生的需求和学习进度，提供个性化的教学方案，提高了教学效果。在商业领域，电子商务平台和移动支付技术使购物更加便捷和高效。

然而，数字创新也带来了挑战。如何在享受数字便利的同时，保障个人信息的安全，是一个亟待解决的问题。此外，随着技术的快速发展，传统行业和岗位面临被淘汰的风险。我们在拥抱变革的 同时，也需要采取有效措施应对挑战，确保数字创新带来更多的机遇和福祉。

Translation:

Digital innovation is rapidly shaping the way we live and work. From artificial intelligence and big data, to the Internet of Things and mobile payments, each technology is driving social transformation. Not only does digital innovation enhance efficiency, it also provides novel solutions to problems that traditional approaches have been unable to address.

In the educational sector, online learning platforms and virtual classrooms have broken down the barriers of time and space, enabling more people to access high-quality educational resources. Educational institutions can leverage big data analytics to gain a better understanding of students’ needs and learning progress, deliver personalized teaching plans and better pedagogical outcomes. In the business sector, e-commerce platforms and mobile payment technologies have made shopping more convenient and efficient.

However, digital innovation is not without its challenges. Safeguarding personal information in an increasingly digitalized environment remains a pressing concern. Moreover, the rapid advancement of technology threatens certain traditional industries and jobs. While embracing the change, we must also take effective measures to address these challenges and ensure that digital innovation brings about more opportunities and greater well-being.

**Appendix B SRL Questionnaire**

This questionnaire asks about how you practice interpreting. It is anonymous and is used for research purposes only. There are no right or wrong answers. Please answer according to what you actually do.

1 = Never    2 = Rarely    3 = Sometimes    4 = Often    5 = Always

1. Before practicing, I review the assessment criteria and clarify what I need to achieve.

2. I set specific improvement goals for a session based on the feedback I received previously.

3. Before interpreting, I anticipate the difficulties in the material and work out how to deal with them.

4. While interpreting, I check whether my wording and grammar are accurate.

5. While interpreting, I monitor my delivery, noticing unnecessary fillers and pauses.

6. When I notice a problem, I correct it on the spot (e.g. self-correction, rephrasing).

7. While interpreting, I judge whether the message I convey is complete and faithful to the source.

8. While interpreting, I attend to whether my output is logically coherent.

9. While interpreting, I have almost no capacity left to attend to how well I am doing.

10. Before looking at the feedback, I first judge my own performance.

11. I go through the individual rating categories to see which aspects I need to improve.

12. I look at the reference rendition and compare it with my own output.

13. I look into the causes of my problems (e.g. comprehension, memory, expression, insufficient preparation).

14. I re-practice the material or the segments in which I had problems, based on the feedback.

15. I draw lessons from a session and work out how to do better next time.

16. Once I have seen the score, I usually do not go through the feedback in detail.

1 = Strongly disagree    2 = Disagre    3 = Neutral    4 = Agree    5 = Strongly agree

17. I am willing to put extra time and effort into interpreting practice, even when the material is difficult.

18. If I invest enough effort, my interpreting will improve.

19. I am confident that I can cope with content I have not prepared for.

20. When I perform badly, I feel like giving up practicing.

## Appendix C Semi-structured Interview Protocol

Interviews were conducted in Chinese (participants' L1). the English version below is a translation for reference. The eight main questions were asked of all participants; the probes were used flexibly and were not necessarily all asked.

Participant information:

Code (S____); gender; year of study; years of interpreting training; language direction; length of system use (weeks); average sessions per week; interview date and mode.

Q1. What preparation do you usually do before each interpreting practice session?

What exactly do you do, and how much time does it take?

Did these habits exist before you used the system, or develop afterwards?

Q2. How do you set goals for a practice session? Could you give an example?

Are your goals more outcome-oriented (e.g., "score above 80") or process-oriented (e.g., "control my pauses")?

If you don't reach a goal, how do you adjust it next time?

Q3. Do you monitor your own performance while you are practicing? If so, how?

What do you pay attention to while interpreting (omissions, coherence, fluency, pauses, timing)?

When you notice a problem mid-task, what do you do at that moment?

Q4. Compared with when you first started using the system, has your practice frequency changed? Why?

Roughly how many sessions per week then and now?

Apart from frequency, has the way you practice or your level of engagement changed?

Q5. When you look at the automatic scores after a session, which indicators or types of information do you pay most attention to? Why?

Do you look at the overall score or the sub-scores first, and why?

Do the scores match your own judgement? What do you do when they don't?

Q6. Do you actively compare your own recording with the reference translation and analyse the causes of your problems? In what ways do you find the scores and the reference translation helpful?

What does this comparison process look like in practice?

How do you use the reference translation? As a model to imitate, one possible version, or a standard answer?

Q7. How do you feel when the material is difficult or your score is lower than expected? How do you adjust your attitude or practice strategies?

Could you describe a specific session that frustrated you, and what you did next?

How do you explain a poor score to yourself (effort, ability, material difficulty, scoring accuracy)?

Q8. Overall, what effect has using the system had on your initiative, confidence, and interest in interpreting practice?

Which of the three has been affected most? Could you give an example?

Have there been any negative effects (e.g., score anxiety, over-reliance on the system)?

**Appendix D Coding Framework: Themes, Categories, and Codes**

| **Theme / Category / Code** | **Operational Definition** |
|---|---|
| 1. Pre-learning Planning | Cognitive and strategic activity occurring prior to engagement with the task, including task analysis, goal setting, strategy selection, and allocation of time and resources. |
| 1.1 Goal Orientations | The content, source and specificity of the targets students set before practicing. |
| 1.1.1 Feedback-Driven Target Setting | |
| 1.1.2 Material Orientation | |
| 1.1.3 Outcome-Focused Perception | |
| 1.1.4 Protective Goal Setting | |
| 1.1.5 Target Setting | |
| 1.1.6 Topic Orientation | |
| 1.1.7 Vague Goal Orientation | |
| 1.2 Temporal Scheduling | How much time was set aside for preparation and when it was fitted in. |
| 1.2.1 Time Allocation | |
| 1.3 Mental Preparation | Cognitive and attentional readying carried out immediately before the task. |
| 1.3.1 Cognitive Priming | |
| 1.3.2 Focus Shift | |
| 1.3.3 Perceived Task Control | |
| 1.3.4 Selective Attention Setting | |
| 1.3.5 Warm-up Practice | |
| 1.4 Planning Impediments | Conditions that displaced, fragmented or replaced forward-looking planning. |

| | |
|---|---|
| 1.4.1 Retrospective Error Attribution | |
| 1.4.2 Fragmented Practice Planning | |
| 1.4.3 Inconsistent Planning | |
| 1.4.4 Ineffective Feedback | |
| 1.4.5 Time Allocation Constraint | |
| 2. Execution and Monitory | Deployment of learning strategies during the task, together with concurrent tracking of comprehension and progress and real-time adjustment of approach. |
| 2.1 Attentional Regulation | Allocation of limited attention across the four dimensions during interpreting. |
| 2.1.1 Feedback-driven Focus | |
| 2.1.2 Selective Attention | |
| 2.2 Strategic Adaptation | Changes to interpreting and practice procedures made in response to feedback. |
| 2.2.1 Remedial Strategy Execution | |
| 2.2.2 Strategy Adjustment | |
| 2.2.3 Strategy Selection | |
| 2.3 Effort Calibration | How much effort was invested, withheld or redistributed, and the capacity limits on doing so. |
| 2.3.1 Cognitive Overload | |
| 2.3.2 Efficiency Priority | |
| 2.3.3 Effort Regulation | |
| 2.3.4 External Time Pressure | |
| 2.3.5 Increased Effort Investment | |
| 2.3.6 Increased Practice | |

| | |
|---|---|
| 2.4 Real-Time Awareness | Awareness of one's own performance while or just after producing it, and its baseline contrast. |
| 2.4.1 Passivity in Traditional Practice | |
| 2.4.2 Real-Time Error Awareness | |
| 2.4.3 System-Triggered Monitoring | |
| 2.4.4 Unsystematic Monitoring | |
| 3. Reflection and Evaluation | Retrospective appraisal of process and outcomes after the task, including self-judgement against personal or external standards, causal attribution of success and failure, and inferences drawn for subsequent tasks. |
| 3.1 Diagnostic Analytics | Use of scores, sub-scores, transcripts and annotations to identify and name problems. |
| 3.1.1 Blind Spot Identification | |
| 3.1.2 Diagnostic Signal | |
| 3.1.3 Diagnostic Error Attribution | |
| 3.1.4 Problem Diagnosis | |
| 3.1.5 Weakness Tracking | |
| 3.2 Feedback Processing | How the different feedback outputs were valued, trusted and acted upon. |
| 3.2.1 Actionable Feedback Preference | |
| 3.2.2 Black-Box Feedback | |
| 3.2.3 Feedback Use | |
| 3.2.4 Instant Correction | |
| 3.3 Evaluative Comparison | Comparisons through which performance was judged: against a total, a past attempt, or a model. |

| | |
|---|---|
| 3.3.1 Overall Score Focus | |
| 3.3.2 Selective Inspection | |
| 3.3.3 Temporal Comparison | |
| 3.3.4 Textual Comparison | |
| 3.3.5 Threshold Evaluation | |
| 3.4 Meta-Cognitive Integration | Whether inspection was converted into durable knowledge or causal understanding. |
| 3.4.1 Knowledge Internalization | |
| 3.4.2 Task-Specific Reflection | |
| 4. Emotional Motivation | Affective and motivational states reported in relation to the task, and the deliberate strategies used to generate, sustain, or down-regulate them. |
| 4.1 Affective Regulation | Emotions elicited by scoring and visibility, and the strategies used to manage them. |
| 4.1.1 Anxiety Reduction | |
| 4.1.2 Emotion Regulation | |
| 4.1.3 Emotional Relief | |
| 4.1.4 Evaluative Anxiety | |
| 4.1.5 Negative Affect | |
| 4.1.6 Performance Anxiety | |
| 4.1.7 Shame Avoidance | |
| 4.2 Self-Efficacy Dynamics | Beliefs about one’s own capability, and the sense of control over practice. |
| 4.2.1 Fluctuating Self-Efficacy | |
| 4.2.2 Increased Agency | |

| | |
|---|---|
| 4.2.3 Low Self-Efficacy | |
| 4.2.4 Uncertain Self-Efficacy | |
| 4.3 Motivational Drivers | The sources of, and limits on, students' willingness to practice. |
| 4.3.1 High Perceived Task Difficulty | |
| 4.3.2 Intrinsic Motivation | |
| 4.3.3 Low Intrinsic Motivation | |
| 4.3.4 Performance-Driven Motivation | |
| 4.3.5 Progress-Driven Interest | |
| 4.3.6 Unchanged Intrinsic Motivation | |
| 4.4 Technological Orientations | Global appraisal of the platform as a learning tool. |
| 4.4.1 Technology Acceptance | |
| 4.4.2 Tool Neutrality | |
